# One-Step ^18^F-Labeling and Preclinical Evaluation of Prostate-Specific Membrane Antigen Trifluoroborate Probes for Cancer Imaging

**DOI:** 10.2967/jnumed.118.216598

**Published:** 2019-08

**Authors:** Hsiou-Ting Kuo, Mathieu L. Lepage, Kuo-Shyan Lin, Jinhe Pan, Zhengxing Zhang, Zhibo Liu, Alla Pryyma, Chengcheng Zhang, Helen Merkens, Aron Roxin, David M. Perrin, François Bénard

**Affiliations:** 1 BC Cancer, Vancouver, British Columbia, Canada; 2 Department of Radiology, University of British Columbia, Vancouver, British Columbia, Canada; and; 3 Chemistry Department, University of British Columbia, Vancouver, British Columbia, Canada

**Keywords:** PSMA, ^18^F-trifluoroborate, ^18^F labeling, positron emission tomography, prostate cancer

## Abstract

After the identification of the high-affinity glutamate-ureido scaffold, the design of several potent ^18^F- and ^68^Ga-labeled tracers has allowed spectacular progress in imaging recurrent prostate cancer by targeting the prostate-specific membrane antigen (PSMA). We evaluated a series of PSMA-targeting probes that are ^18^F-labeled in a single step for PET imaging of prostate cancer. **Methods:** We prepared 8 trifluoroborate constructs for prostate cancer imaging, to study the influence of the linker and the trifluoroborate prosthetic on pharmacokinetics and image quality. After 1-step labeling by ^19^F–^18^F isotopic exchange, the radiotracers were injected in mice bearing LNCaP xenografts, with or without blocking controls, to assess specific uptake. PET/CT images and biodistribution data were acquired at 1 h after injection and compared with ^18^F-DCFPyL on the same mouse strain and tumor model. **Results:** All tracers exhibited nanomolar affinities, were labeled in good radiochemical yields at high molar activities, and exhibited high tumor uptake in LNCaP xenografts with clearance from nontarget organs. Most derivatives with a naphthylalanine linker showed significant gastrointestinal excretion. A radiotracer incorporating this linker with a dual trifluoroborate-glutamate labeling moiety showed high tumor uptake, low background activity, and no liver or gastrointestinal track accumulation. **Conclusion:** PSMA-targeting probes with trifluoroborate prosthetic groups represent promising candidates for prostate cancer imaging because of facile labeling while affording high tumor uptake values and contrast ratios that are similar to those obtained with ^18^F-DCFPyL.

The prostate-specific membrane antigen (PSMA), a transmembrane metalloenzyme (1), is highly overexpressed in prostate cancer and tumor-associated neovasculature (2). PSMA-targeting constructs have been designed and evaluated as imaging agents for visualizing prostate cancer, most notably by PET (3–6). The diamino acid glutamate-ureido is commonly used for PSMA targeting because of synthetic ease, rapid pharmacokinetics, and high contrast ratios (7). ^68^Ga-PSMA-11 is currently the most commonly used radioligand for prostate cancer imaging (8,9). The short half-life of ^68^Ga (68 min) generally restricts distribution to clinics that are close to a ^68^Ge–^68^Ga generator, which itself limits daily production to 2–4 clinical doses unless direct production using a more complex solid-target apparatus is implemented (10). In contrast, ^18^F has several advantages, including a longer half-life (109.8 min); higher spatial resolution than ^68^Ga due to its short positron range; and on-demand, scalable production of ^18^F-fluoride ions up to a few hundred gigabecquerels (11).

To this effect, ^18^F-labeled PSMA-targeting radiotracers such as ^18^F-DCFPyL (12) and ^18^F-PSMA-1007 (13) have been introduced in clinical studies. We sought to explore a new ^19^F–^18^F isotope exchange reaction on organotrifluoroborate (RBF_3_^−^) groups to develop PSMA-targeting radiotracers. With this approach, a precursor is converted into a radiotracer of identical chemical composition. Isotope exchange labeling of RBF_3_^−^ groups provides good activity yields (15%–60%) and high molar activity values (≥75 GBq/μmol) (14,15). This method has been successfully applied to several ^18^F-RBF_3_^−^–based radiotracers (15–23).

We report the synthesis, radiolabeling, and PET imaging of radiotracers based on the glutamate-ureido-lysine scaffold bearing RBF_3_^−^ radioprosthetic groups (compounds **1–8**, Fig. 1). We measured their binding affinity toward PSMA and LogD_7.4_ values and then acquired PET images and ex vivo biodistribution data in mice bearing PSMA-expressing LNCaP prostate cancer xenografts. These results were compared with those of ^18^F-DCFPyL, a clinically emergent ^18^F-labeled tracer for prostate cancer imaging.

**FIGURE 1.**
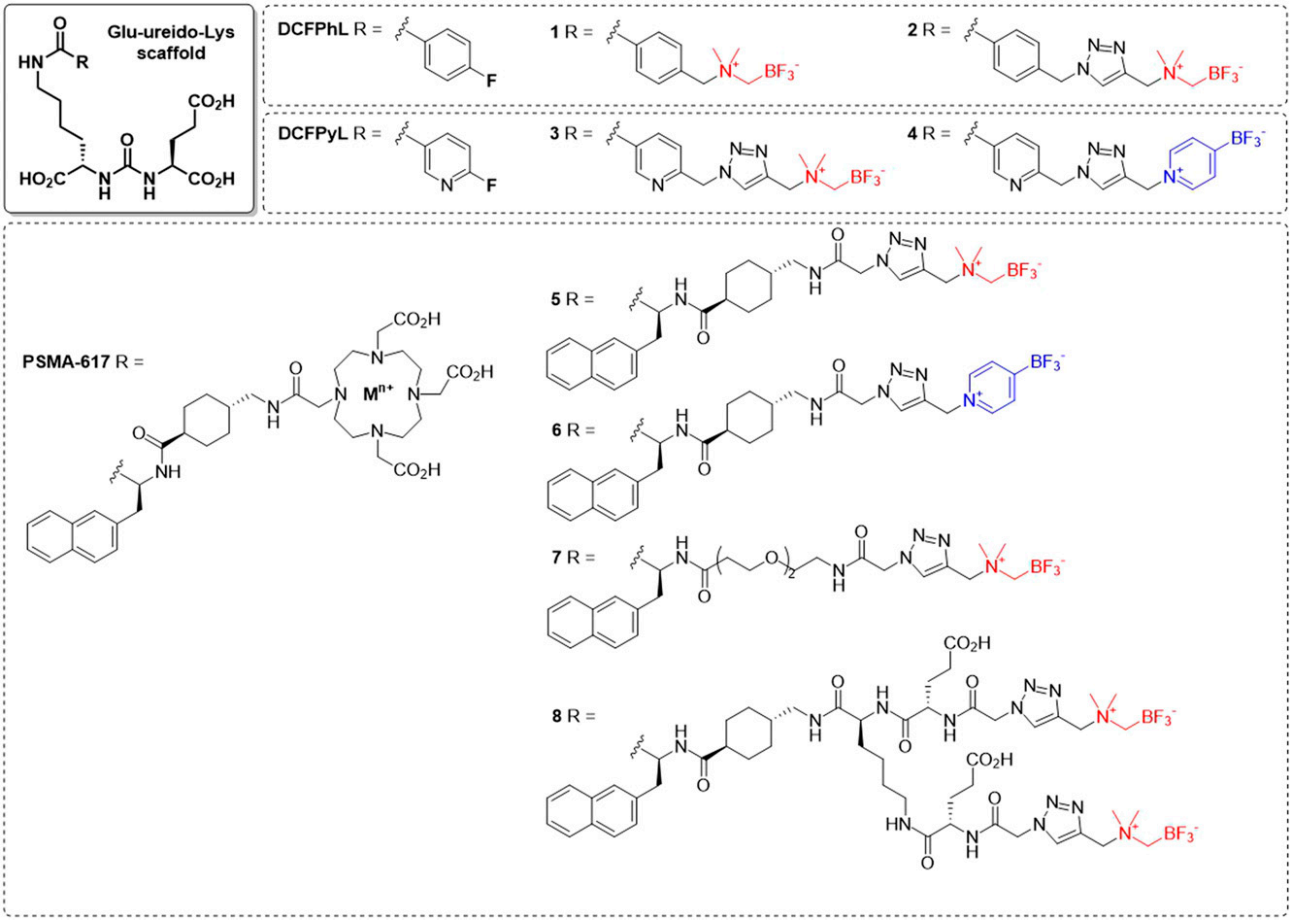
Trifluoroborate probes resembling scaffolds of DCFPhL, DCFPyL, and PSMA-617. In red: AMBF_3_ prosthetic; in blue: pyrBF_3_ prosthetic.

## MATERIALS AND METHODS

### Synthesis of Trifluoroborate Probes and Radiosynthesis

^18^F-DCFPyL was prepared following literature procedures (24). Precursors for tracers **1–8** were synthesized as described in the supplemental data section (available at http://jnm.snmjournals.org) to give azide-bearing precursors (6,18,21,24–28), which were conjugated to previously reported alkyne-bearing RBF_3_^−^ (19). After conjugation, the final trifluoroborate conjugate was purified by high-performance liquid chromatography (HPLC), and purity was confirmed by electrospray ionization–mass spectrometry. Representative crude and quality control HPLC traces are provided in the supplemental data section. ^18^F-**1–8** were labeled via previously reported procedures (15,29). Briefly, 30–40 GBq of no-carrier-added ^18^F-fluoride were trapped on a QMA light cartridge and eluted with 0.9% saline or phosphate-buffered saline (typically 100 μL) directly into a septum-sealed falcon tube containing 80–100 nmol of precursors **1–8** dissolved in 50:50 dimethylformamide:water containing 1 M pyridazinium-HCl buffer (pH 2.5). The reaction was heated to 80°C, and a vacuum was applied to reduce the reaction volume. After 15–20 min, the reaction was quenched by addition of 2 mL of 40 mM ammonium formate or phosphate-buffered saline, and the contents were purified by semipreparative HPLC. Radiochemical purity was confirmed by HPLC analysis using an analytic RP-C18 column with gradients of acetonitrile and water (both containing 0.1% trifluoroacetic acid). Measurements of molar activity values were based on standard curve analysis.

### Cell Culture

The LNCaP cell line was obtained from ATCC (LNCaP clone FGC, CRL-1740). The cells were cultured in RPMI 1640 medium supplemented with 10% fetal bovine serum, penicillin (100 U/mL), and streptomycin (100 μg/mL) at 37°C in a MCO-19AIC (Panasonic Healthcare) humidified incubator containing 5% CO_2_. Cells grown to 80%–90% confluence were then washed with sterile phosphate-buffered saline (1 × phosphate-buffered saline, pH 7.4) and trypsinized. The collected cell number was counted with a Bal Supply 202C laboratory counter.

### In Vitro Competition Binding Assay

Inhibition constants (K_i_) of **1–8** and DCFPyL to PSMA were measured by in vitro competition binding assays using ^18^F-DCFPyL as the radioligand. LNCaP cells were plated onto a 24-well poly-d-lysine coated plate for 48 h (400,000/well). Growth medium was removed and replaced with 4-(2-hydroxyethyl)-1-piperazineethanesulfonic acid (HEPES) buffered saline (50 mM HEPES, pH 7.5, 0.9% sodium chloride) After 1 h, ^18^F-DCFPyL (0.1 nM) was added to each well (in triplicate) containing varied concentrations (0.5 mM–0.05 nM) of tested compounds (DCFPyL, **1–8**). Nonspecific binding was determined in the presence of 10 μM unlabeled DCFPyL. The assay mixtures were incubated for 1 h at 37°C with gentle agitation followed by 2 washes with cold HEPES buffered saline. A trypsin solution (0.25%, 400 μL) was then added to each well to harvest the cells. Radioactivity was measured by γ-counting, and K_i_ values were calculated using the “1 site—fit K_i_” built-in model in Prism 7 (GraphPad). The dissociation constant value for ^18^F-DCFPyL, used for K_i_ determination, was 0.49 nM, as previously measured by saturation assays using LNCaP cells (30).

### Distribution Coefficient (LogD_7.4_) Measurements

LogD_7.4_ values were measured using the shake flask method. Briefly, an aliquot of ^18^F-labeled tracer was added to a vial containing 2.5 mL of *n*-octanol and 2.5 mL of phosphate buffer (0.1 M, pH 7.4). The mixture was vortexed for 2 min and then centrifuged at 3,000*g* for 10 min. A sample of the *n*-octanol (0.1 mL) and buffer (0.1 mL) layers was counted using a γ-counter. Values of LogD_7.4_ were calculated using the following equation: LogD_7.4_ = log_10_ [(counts in *n*-octanol phase)/(counts in buffer phase)].

### PET/CT Imaging and Biodistribution Studies

Imaging and biodistribution experiments were performed using NODSCID IL2RγKO male mice. All experiments were conducted according to the guidelines established by the Canadian Council on Animal Care and approved by the Animal Ethics Committee of the University of British Columbia. Mice were anesthetized by inhalation with 2% isoflurane in oxygen and implanted subcutaneously with 1 × 10^7^ LNCaP cells behind the left shoulder. The mice were imaged or used in biodistribution studies once the tumor reached 5–8 mm in diameter (5–6 wk).

PET imaging experiments were conducted using an Inveon preclinical PET/CT scanner (Siemens). Compounds ^18^F-**1,2,3,5,7,** and **8** were formulated in 10% ethanol/normal saline, whereas ^18^F-**4** and **6** were formulated in 10% ethanol/phosphate-buffered saline. Each tumor-bearing mouse was injected with 6–8 MBq of ^18^F-**1–8** or ^18^F-DCFPyL through the tail vein under sedation (2% isoflurane in oxygen). For blocking controls, the mice were coinjected with DCFPyL (0.5 mg). After injection, the mice were allowed to recover and roam freely in their cage. After 50 min, the mice were sedated by 2% isoflurane inhalation and positioned in the scanner. A CT scan was performed first for localization and attenuation correction. This was followed by a 10-min static PET scan. The mice were kept warm by a heating pad during image acquisition. PET images were reconstructed using the IAW software (Siemens), using 2 iterations of the ordered-subset expectation maximization algorithm followed by 18 iterations of the maximum a posteriori algorithm.

For biodistribution and blocking studies, the mice were injected with 1–3 MBq of radiotracer. At 60 min, the mice were anesthetized with 2% isoflurane inhalation and euthanized by CO_2_ inhalation. Blood was withdrawn by cardiac puncture, and the organs and tissues of interest were collected, weighed, and counted using an automatic γ-counter (PerkinElmer). Uptake values were expressed as the percentage of the injected dose per gram of tissue (%ID/g).

### Statistical Analysis

A standard 1-way ANOVA was performed to determine whether statistically significant differences in tumor uptake occurred between radiotracers. Each radiotracer was compared with ^18^F-DCFPyL using the Dunnett test (a many-to-one *t* test comparison). This analysis was also performed for kidney and blood activity and for tumor-to-blood and tumor-to-muscle ratios. Reported *P* values were adjusted for multiple comparisons. The analysis was performed using Prism 8 (GraphPad).

## RESULTS

### Radiolabeling

Starting with 37 GBq of ^18^F-fluoride, **1**–**8** (80–100 nmol) were successfully labeled within 25 min, with activity yields ranging from 4% to 16% (Table 1) at high molar activities (≥56 GBq/μmol). In all cases, the radiochemical purity was at least 99% by analytic HPLC. Compounds bearing the pyridine-trifluoroborate (pyrBF_3_) prosthetic (**4, 6**) were labeled in higher yields and molar activities than conjugates bearing the ammoniomethyl-trifluoroborate (AMBF_3_) prosthetic (**3, 5**). Although HPLC was used to isolate tracers at greater than or equal to 99% radiochemical purity, HPLC purification can be avoided: ^18^F-**6** was purified on a Sep-Pak C_18_ cartridge according to reported procedures (29). In that case, the radiochemical purity was at least 95%. In addition, we deliberately labeled **2** at lower molar activity; starting with 37 GBq of no-carrier-added ^18^F-fluoride and 1 μmol of precursor; ^18^F-**2** was obtained in 34% activity yield at 13.3 GBq/μmol (Table 2).

**TABLE 1:** Activity Yield, Molar Activity, Partition Coefficient, and Binding Affinity of ^18^F-Labeled PSMA Radiotracers

Tracer	Activity yield* (%, isolated)	Molar activity^†^ (GBq/μmol)	LogD_7.4_ (*n* = 3)	K_i_ (nM) (*n* = 3)
^18^F-DCFPyL	12 ± 3 (*n* = 7)	118 ± 37 (*n* = 7)	−3.12 ± 0.22	2.0 ± 0.8
^18^F-**1**	7 ± 4 (*n* = 4)	70 ± 19 (*n* = 4)	−3.43 ± 0.35	14.4 ± 2.7
^18^F-**2**	4 ± 2 (*n* = 3)	89 ± 26 (*n* = 3)	−4.26 ± 0.04	11.8 ± 0.9
^18^F-**3**	5 ± 1 (*n* = 3)	56 ± 15 (*n* = 3)	−4.01 ± 0.14	25.9 ± 5.7
^18^F-**4**	16 ± 2 (*n* = 3)	148 ± 89 (*n* = 3)	−3.34 ± 0.02	27.6 ± 3.8
^18^F-**5**	13 ± 10 (*n* = 2)	137 ± 22 (*n* = 2)	−3.52 ± 0.21	1.14 ± 0.26
^18^F-**6**	15 ± 2 (*n* = 6)	278 ± 73 (*n* = 6)	−2.28 ± 0.01	1.90 ± 0.68
^18^F-**7**	10 ± 5 (*n* = 3)	92 ± 22 (*n* = 2)	−3.24 ± 0.03	16.5 ± 5.5
^18^F-**8**	7 ± 6 (*n* = 3)	211 ± 48 (*n* = 3)	−3.58 ± 0.36	0.22 ± 0.01

* Activity yields are reported at end of synthesis (1 h for DCFPyL, 40 min for **1–8**) (with no correction for decay).

† Molar activities are reported at time of quality control injection, shortly after end of synthesis.

**TABLE 2:** Changes in Activity Yield and Molar Activity with Higher Quantities of Precursor Material

Tracer	Activity yield (%)	Molar activity (GBq/μmol)
^18^F-**2** from 100 nmol (*n* = 3)	4 ± 2	89 ± 26
^**1**8^F-**2** from 1000 nmol (*n* = 2)	34 ± 9	13.3 ± 0.74
Change	× 8.5	÷ 6.7

### Binding Assays

**FIGURE 2.**
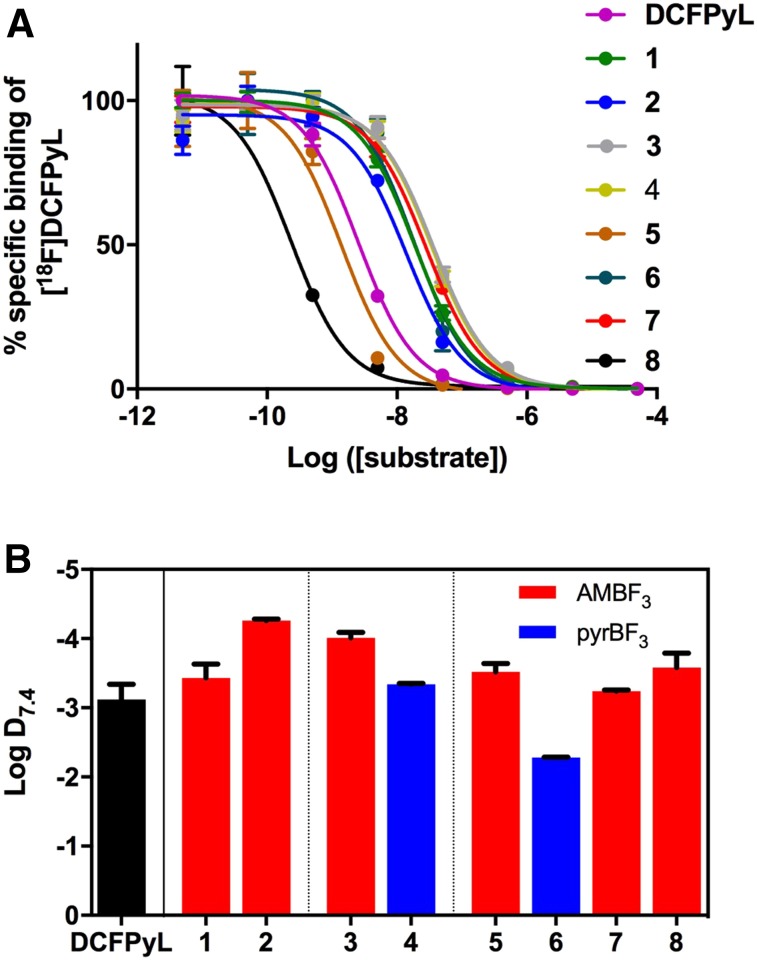
(A) Competitive inhibition curves of DCFPyL and **1–8**. (B) Values of LogD_7.4_ for DCFPyL and **1–8** (error bars reflect SD).

We determined K_i_ via in vitro competition binding assays using LNCaP cells and ^18^F-DCFPyL as the radioligand (Fig. 2A). The K_i_ value for DCFPyL was 2.0 ± 0.8 nM, consistent with the value previously reported by Chen et al. (1.1 ± 0.1 nM) (31). Probes **1**–**4** and **7** had K_i_ values in the 10–30 nM range, whereas **5** and **6** had up to 10-fold better affinities, comparable to that measured for DCFPyL. Probe **8** showed excellent binding affinity to PSMA, with a K_i_ value of 0.22 ± 0.01 nM (Table 1).

### LogD_7.4_

All compounds but **6** had LogD_7.4_ values similar to ^18^F-DCFPyL (Figure 2B and Table 1). Using pyrBF_3_ instead of AMBF_3_ as the prosthetic group decreased hydrophilicity in **4** and **6** compared with **3** and **5**, respectively. Compound **6** was the most lipophilic compound of the series.

### PET/CT Imaging and Biodistribution

**FIGURE 3.**
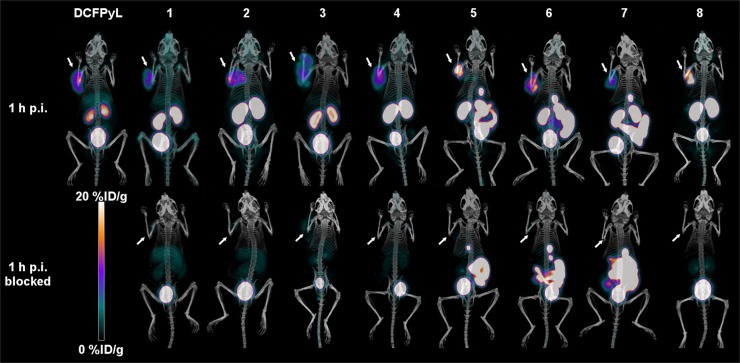
PET/CT images (maximum-intensity projections) of LNCaP tumor–bearing mice at 1 h after injection, with and without blocking by coinjection of unlabeled DCFPyL. Arrows locate tumors.

Imaging ^18^F-DCFPyL confirmed good tumor uptake and fast clearance (31). Similarly, ^18^F-**1**–**8** showed significant tumor uptake in LNCaP xenografts, which was blocked by coinjection of unlabeled DCFPyL (Fig. 3), thus confirming the specificity of tumor uptake for PSMA. All images also showed high, specific kidney uptake along with urinary excretion. Bone accumulation was negligible for all radiotracers. The blocking agent caused significantly lower tumor and kidney uptake values for all compounds.

**FIGURE 4.**
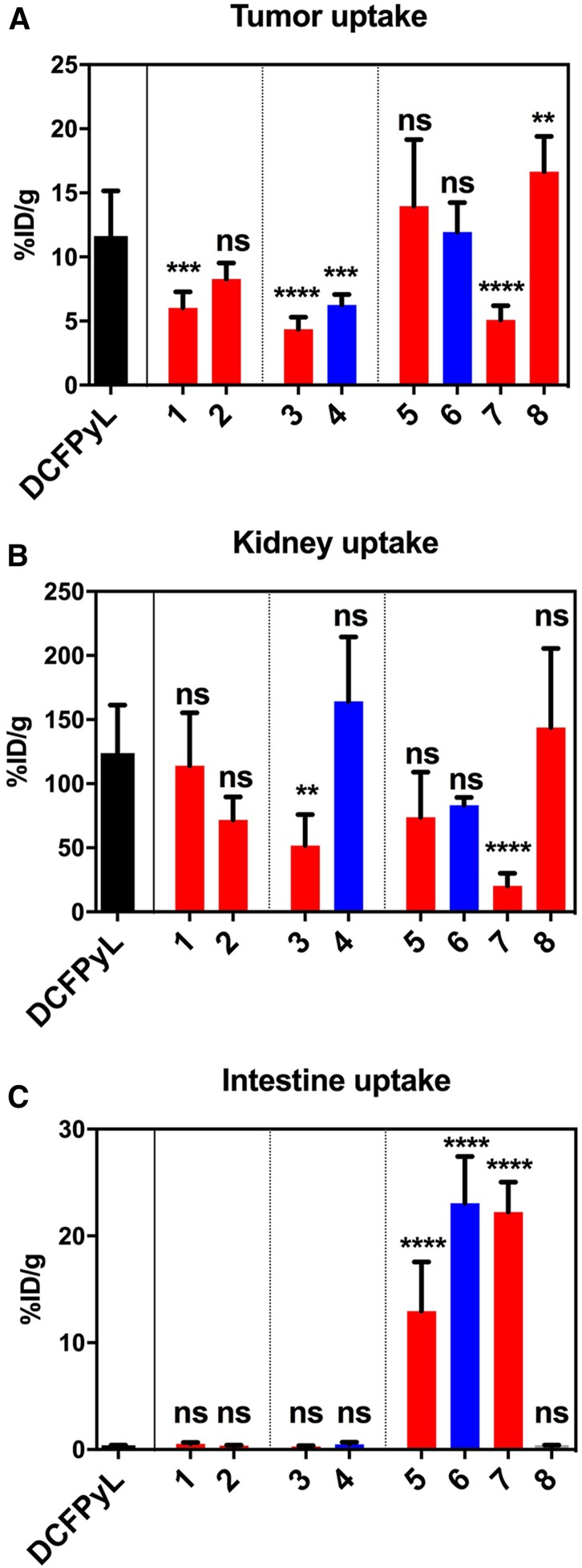
Uptake values for tumor (A), kidney (B), and intestine (C) for compounds **1–8** and DCFPyL; in black: DCFPyL; in red: AMBF_3_ derivatives; in blue: pyrBF_3_ derivatives (error bars reflect SD values, significance of differences with ^18^F-DCFPyL indicated at top of bars: **P < 0.01; ****P < 0.0001; ns = not significant). Full data available in Supplemental Data section.

**FIGURE 5.**
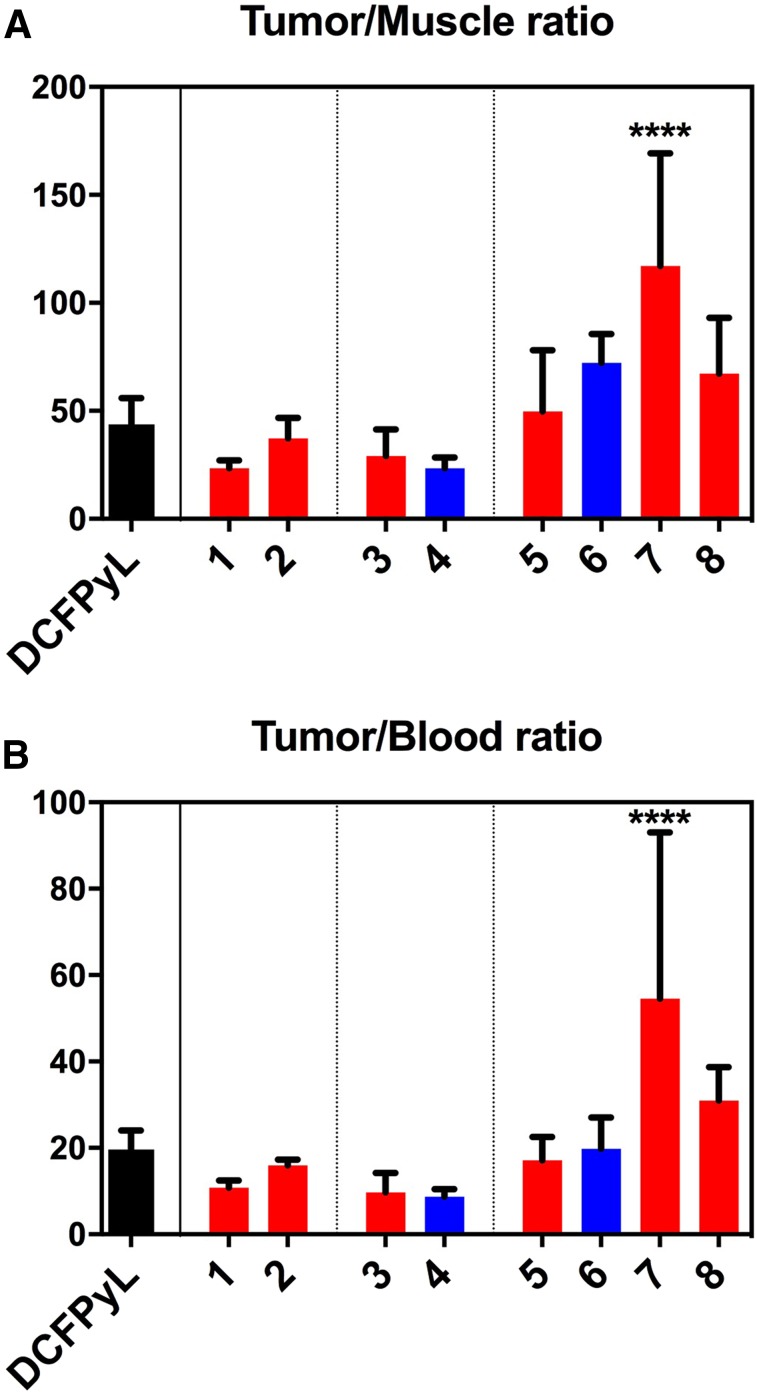
Contrast ratios (tumor-to-muscle in A and tumor-to-blood in B) for compounds **1–8** and DCFPyL at 1 h after injection; in black: DCFPyL; in red: AMBF_3_ derivatives; in blue: pyrBF_3_ derivatives (error bars reflect SD values, significance of differences with ^18^F-DCFPyL indicated at top of bars: ****P < 0.0001).

**FIGURE 6.**
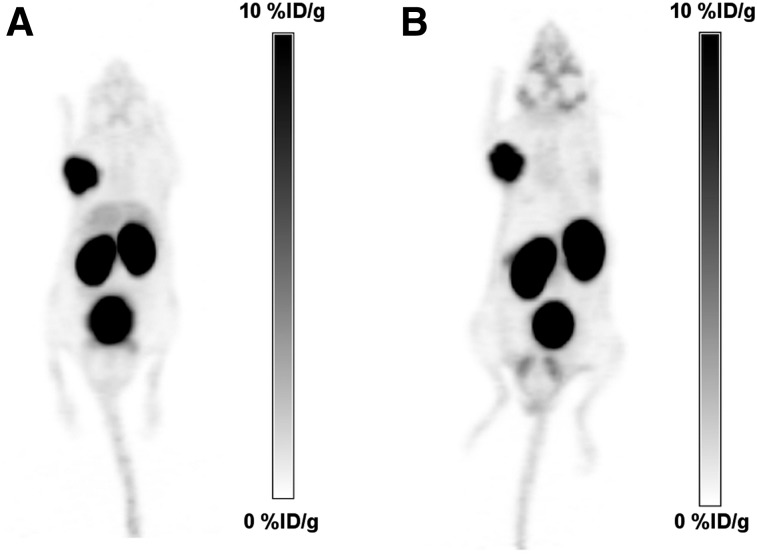
PET/CT images (maximum-intensity projections in black on white to display background activity), comparing ^18^F-DCFPyL (A) with ^18^F-**8** (B), showing similar image contrast with lower liver accumulation for compound **8**. Maximum of scale corresponds to 10 %ID/g for both radiotracers.

The tracers based on a naphthylalanine-tranexamic acid linker (**5** and **6**) displayed tumor uptake values of 13.7 ± 5.2 %ID/g and 11.9 ± 2.3 %ID/g, respectively. ^18^F-**1, 2, 3, 4,** and **7** had uptake values of 6.0 ± 1.2, 8.3 ± 1.3, 4.4 ± 0.95, 6.3 ± 0.8, and 5.1 ± 1.1 %ID/g, respectively. Compounds ^18^F-**1, 3, 4,** and **7** had lower tumor uptake than ^18^F-DCFPyL (Fig. 4A). Compound **8** had high tumor uptake (16.7 ± 2.7 %ID/g). No statistically significant differences were observed between compounds **2, 5,** and **6** and ^18^F-DCFPyL, whereas compound **8** had higher uptake. Compounds **3** and **7** had lower kidney accumulation (Fig. 4B), whereas compounds **5** and **6** had significantly higher intestinal activity than ^18^F-DCFPyL (Fig. 4C). The blocking controls showed that intestinal uptake was not receptor-mediated. The tumor-to-blood and tumor-to-muscle ratios were not statistically different from ^18^F-DCFPyL for any compound except compound **7,** which had higher ratios (Fig. 5). Compound **8,** with 2 AMBF_3_-glutamate motifs, had no significant accumulation in the liver, no hepatobiliary excretion, and low background activity (Fig. 6).

## DISCUSSION

We designed PSMA-targeting radiotracers that combine the advantages of 1-step aqueous ^18^F-labeling afforded by 2 RBF_3_^−^ radioprosthetic groups with certain chemical features found in DCFPyL (or its *C*-analog DCFPhL) (4) and PSMA-617 (32). Since the 3 carboxylates of Glu-ureido-Lys are needed for binding to PSMA, we introduced modifications at the lysine side chain (31,33), off of which we introduced several well-established linkers along with a suitable RBF_3_^−^.

Binding assays confirmed low-nanomolar affinities for compounds **5** and **6,** whereas compound **8** had subnanomolar binding affinity. Compounds **1**–**4** exhibited 10-fold higher affinities than DCFPyL, suggesting that the trifluoroborate prosthetic group may not interact well with the S1 binding pocket in PSMA, which exhibits pronounced affinity for hydrophobic groups (3). Compounds incorporating a naphthylalanine-tranexamic acid motif (**5** and **6**) exhibited improved binding affinities (K_i_ = 1.14 nM and 1.90 nM, respectively) similar to those of DCFPyL (K_i_ = 2.0 nM) and Ga-PSMA-617 (K_i_ = 2.3 nM) (25). Interestingly, the tranexamic acid linker appears to contribute significantly to affinity, as its replacement by a polyethylene glycol 2 spacer (compound **7**) resulted in a higher K_i_. The dual glutamate-BF_3_ motif, introduced to improve the hydrophilicity of the BF_3_ derivatives with a naphthylalanine-tranexamic acid linker, unexpectedly improved the binding affinity of compound **8,** with a K_i_ approximately an order of magnitude better than DCFPyL.

All the RBF_3_^−^-bioconjugates were radiolabeled at activity yields greater than 1.85 GBq at molar activity values of at least 56 GBq/μmol. The pyrBF_3_-modified conjugates showed higher activity yields than those modified with the AMBF_3_, along with higher molar activities, consistent with a report that compared both prosthetic groups in the context of LLP2A-RBF_3_^−^ bioconjugates (19), as well as with the established stabilities of various trifluoroborates, as previously reviewed (34). High molar activities were also achieved with compound **8,** with a dual glutamate-BF_3_ motif. Although imaging and biodistribution studies were performed with HPLC-purified tracers to ensure the highest level of purity, a simple Sep-Pak purification of ^18^F-**6** (<5 min) afforded good radiochemical purity (95%) (supplemental data). This demonstrates potential for HPLC-free labeling where speed is preferred (overall synthesis time < 30 min).

Although radiochemical yields were lower for certain compounds, these syntheses have not been optimized. Notably, yields were dramatically improved by increasing the amount of precursor: the lowest yield (for tracer ^18^F-**2**) was increased more than 8-fold to 34% when using 10 times more precursor. Consequently, the average molar activity of ^18^F-**2** decreased by a similar factor from 89 to 13.3 GBq/μmol. This demonstrates that yields dramatically increase when high molar activity is not critically needed.

To evaluate ^18^F-**1**–**8** for PSMA imaging, PET/CT imaging and biodistribution studies were conducted in mice bearing LNCaP tumor xenografts. Previously, Chen et al. and Harada et al. imaged ^18^F-DCFPyL in different strains of mice bearing different tumor models (31,33), thus complicating a comparison between this work and prior work. Given these discrepancies, we directly compared ^18^F-**1**–**8** with ^18^F-DCFPyL using a single mouse strain and the LNCaP xenograft tumor model, because it expresses PSMA endogenously and is commonly used to evaluate PSMA-targeting radiotracers (25,33).

Imaging and biodistribution studies showed that ^18^F-**1**–**8** and ^18^F-DCFPyL were all retained in tumors and cleared from nontarget tissues and organs, mainly through the renal pathway for compounds ^18^F-**1**–**4** and **8,** and a combination of renal and hepatobiliary clearance for compounds ^18^F-**5**–**7** (Fig. 4). Tumor uptake was higher with ^18^F-**8** than with ^18^F-DCFPyL, a result that might be explained by improved affinity. All compounds showed significant renal uptake, which was blocked by DCFPyL, consistent with the well-documented, high PSMA expression in mouse kidneys (25,31,33,35–38). As with ^18^F-DCFPyL, images acquired with ^18^F-**1**–**4** and **8** showed low uptake in nontarget organs, whereas those acquired with ^18^F-**5**–**7** showed high accumulation in the gallbladder and intestines. Blocking controls showed that this intestinal uptake was not receptor-mediated. Although it is likely that intestinal uptake is due to the hydrophobic naphthylalanine moiety, this was not noted with ^68^Ga- or ^177^Lu-labeled PSMA-617 tracers (32). We presume that the DOTA chelator promotes renal clearance.

Because many radiotracers were compared with ^18^F-DCFPyL, this study did not have statistical power to evaluate small differences between radiotracers. The results confirmed the versatility of RBF_3_^−^ prosthetic groups for ^18^F radiolabeling, and potential strategies to direct radiotracers to favor hepatobiliary or renal clearance.

Renal clearance can be a drawback for prostate cancer imaging, as focal retention in the ureters may be confused with small nodal metastases, and because high bladder activity may obscure the detection of primary prostate tumors or recurrences. Conversely, excessive bowel activity may also be detrimental for detection of small lesions in the pelvis and abdomen. High liver activity, as observed in clinical studies with ^18^F-DCFPyL (12) and ^18^F-PSMA-1007 (13), might impair detection of liver tumors, notably for detection of hepatocellular carcinomas, for which PSMA imaging may be of value (39).

Other ^18^F-labeled PSMA binding radiotracers have recently been reported, notably ^18^F-PSMA-1007 (13,40), among others (41–44). The RBF_3_^−^ radiotracers presented in this article were not directly compared with these compounds. With an excellent binding affinity, high tumor accumulation, and no liver or gastrointestinal excretion, ^18^F-**8** represents an attractive radiopharmaceutical for clinical translation.

## CONCLUSION

We report promising alternatives to current ^18^F- and ^68^Ga-labeled PSMA-targeting agents, as the RBF_3_^−^ prosthetic groups enable a facile, 1-step ^18^F-labeling in aqueous medium. Labeling times could be further reduced to 30 min with a simple Sep-Pak purification. The 1-step labeling by isotope exchange provided for the simple production of a precursor that is chemically identical to the radiolabeled product, simplifying aspects of both production and labeling. These radiotracers were designed to explore the influence of both the spacer and the trifluoroborate prosthetic group. Compound **8,** with a naphthylalanine-tranexamic acid linker and a dual glutamate-BF_3_ moiety designed to enhance hydrophilicity, showed excellent binding affinity and high tumor uptake without liver accumulation or hepatobiliary clearance.

## DISCLOSURE

This work depicts compounds pertaining to patent WO 2017/117687 A1, which entitles certain authors (Hsiou-Ting Kuo, Mathieu Lepage, Jinhe Pan, Zhibo Liu, Aron Roxin, François Bénard, Kuo-Shyan Lin, and David Perrin) to royalties upon licensing. This work was supported by the Michael Smith Foundation for Health Research, the Canadian Cancer Society (grant #704366), and the Canadian Institutes for Health Research (grant #FDN-148465). No other potential conflict of interest relevant to this article was reported.
