# Supplementary material for: One-Step 18F-Labeling and Preclinical Evaluation of Prostate-Specific Membrane Antigen Trifluoroborate Probes for Cancer Imaging
Source: J Nucl Med. 2019 Aug;60(8):1160–6. doi: 10.2967/jnumed.118.216598 (PMC6681697; doi:10.2967/jnumed.118.216598)
Supplement: Supplementary file 1 [file jnm216598SupplementalData.pdf]

COMPARATIVE QC ANALYSIS OF TRACER [^{18}F]6 PURIFIED BY SEMI-PREPARATIVE HPLC OR SEP-PAK C_{18} TRAPPING

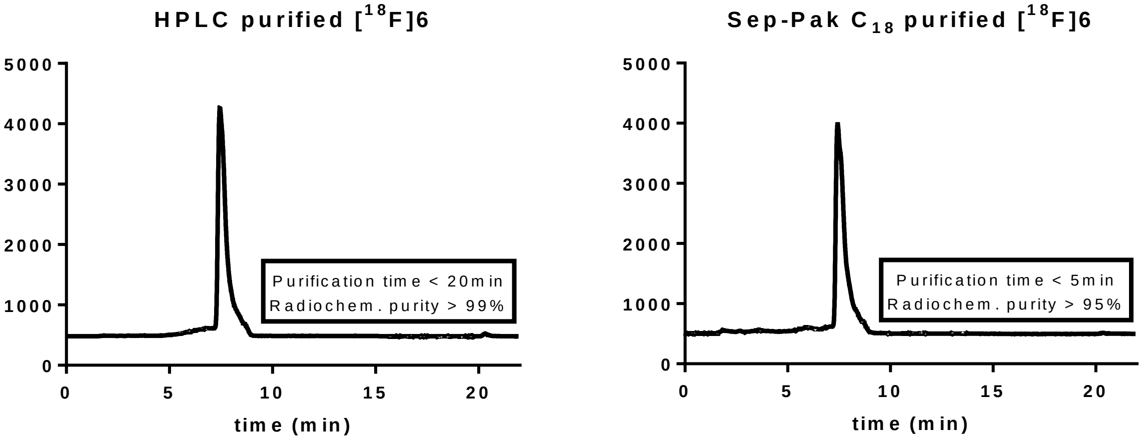

Supplemental Figure 1 – Comparative QC analysis of tracer **[^{18}F]6** purified by semi-preparative HPLC (left) or Sep-Pak C_{18} trapping.

COMPLETE BIODISTRIBUTION STUDIES

Statistical analysis

Statistical analyses were performed using GradPad prism 7. Multiple *t* tests were performed to compare biodistribution in unblocked and blocked mice, multiple comparisons were corrected using the Holm-Sidak method. The difference was considered statistically significant when *p* value was < 0.05.

Supplemental Table 1 – Complete biodistribution study for compounds **[^{18}F]1–3**

	[^{18}F]1		[^{18}F]2		[^{18}F]3	
**Tissue (%ID/g)**	1h n = 8	1h blocked n = 4	1h n = 6	1h blocked n = 4	1h n = 8	1h blocked n = 5
**blood**	0.57 ± 0.15	0.45 ± 0.27	0.52 ± 0.06	0.46 ± 0.24	0.50 ± 0.16	0.30 ± 0.10
**fat**	0.99 ± 0.39	0.14 ± 0.08*	0.73 ± 0.26	0.06 ± 0.01**	0.35 ± 0.15	0.16 ± 0.10
**testes**	0.62 ± 0.15	0.10 ± 0.02***	0.67 ± 0.17	0.08 ± 0.02**	0.30 ± 0.09	0.10 ± 0.03**
**intestine**	0.54 ± 0.11	0.57 ± 0.23	0.33 ± 0.05	0.31 ± 0.02	0.28 ± 0.07	0.23 ± 0.06
**stomach**	0.12 ± 0.05	0.11 ± 0.08	0.10 ± 0.03	0.06 ± 0.02	0.08 ± 0.04	0.06 ± 0.02
**spleen**	2.67 ± 0.98	0.13 ± 0.03**	5.01 ± 0.64	0.09 ± 0.03***	0.84 ± 0.52	0.09 ± 0.05
**liver**	2.90 ± 0.56	3.00 ± 0.53	1.69 ± 0.19	1.76 ± 0.17	1.17 ± 0.28	1.25 ± 0.17
**pancreas**	0.55 ± 0.16	0.11 ± 0.03**	0.33 ± 0.05	0.06 ± 0.01***	0.27 ± 0.13	0.08 ± 0.03
**adrenal**	4.77 ± 1.75	0.35 ± 0.14**	4.65 ± 1.75	0.20 ± 0.05**	1.35 ± 0.52	0.24 ± 0.07**
**kidney**	114.00 ± 41.30	3.54 ± 0.83**	71.70 ± 18.0	2.11 ± 0.19***	51.80 ± 24.10	2.12 ± 0.73*
**lung**	1.37 ± 0.36	0.25 ± 0.06**	1.39 ± 0.17	0.22 ± 0.03***	0.66 ± 0.19	0.30 ± 0.11*
**heart**	0.30 ± 0.06	0.15 ± 0.04*	0.33 ± 0.08	0.09 ± 0.02**	0.19 ± 0.07	0.12 ± 0.06

**tumor**	**6.04** ± 1.24	0.33 ± 0.07***	**8.28** ± 1.25	0.27 ± 0.06***	**4.36** ± 0.95	0.35 ± 0.21***
**muscle**	0.26 ± 0.08	0.13 ± 0.03	0.23 ± 0.04	0.12 ± 0.05*	0.17 ± 0.07	0.09 ± 0.03
**bone**	0.36 ± 0.02	0.30 ± 0.07	0.44 ± 0.09	0.30 ± 0.06	0.20 ± 0.06	0.16 ± 0.06
**brain**	0.04 ± 0.01	0.03 ± 0.01	0.04 ± 0.01	0.02 ± 0.00*	0.03 ± 0.01	0.02 ± 0.00
**T/M**	23.43 ± 3.71	2.63 ± 1.10***	37.30 ± 9.53	2.66 ± 1.60**	29.00 ± 12.40	3.54 ± 1.43*
**T/B**	10.82 ± 1.64	0.91 ± 0.44***	15.95 ± 1.37	0.77 ± 0.48***	9.68 ± 4.53	1.17 ± 0.70*
**T/K**	0.07 ± 0.06	0.10 ± 0.03	0.12 ± 0.04	0.13 ± 0.02	0.11 ± 0.08	0.16 ± 0.05

Significance of differences between unblocked and blocked groups: $^{*}p < 0.05$; $^{**}p < 0.01$; $^{***}p < 0.001$.

Supplemental Table 2 – Complete biodistribution study for compounds **[^{18}F]4–6**.

Tissue (%ID/g)	**[^{18}F]4** 1h n = 7	1h blocked n = 4	**[^{18}F]5** 1h n = 6	1h blocked n = 4	**[^{18}F]6** 1h n = 5	1h blocked n = 4
**blood**	0.74 ± 0.15	0.24 ± 0.11**	0.89 ± 0.42	0.44 ± 0.02	0.68 ± 0.26	1.64 ± 2.58
**fat**	1.05 ± 0.49	0.04 ± 0.03*	0.83 ± 0.33	0.16 ± 0.06	0.38 ± 0.14	0.06 ± 0.02*
**testes**	0.67 ± 0.27	0.08 ± 0.03*	0.74 ± 0.55	0.24 ± 0.05	0.33 ± 0.05	0.14 ± 0.04**
**intestine**	0.48 ± 0.22	0.18 ± 0.04	12.96 ± 4.61	12.36 ± 0.55	23.05 ± 4.39	24.50 ± 4.86
**stomach**	0.15 ± 0.03	0.06 ± 0.02**	0.37 ± 0.45	0.12 ± 0.10	1.17 ± 1.35	0.88 ± 0.41
**spleen**	3.36 ± 1.08	0.13 ± 0.06**	3.21 ± 1.73	0.21 ± 0.02	1.77 ± 0.70	0.18 ± 0.10*
**liver**	1.28 ± 0.18	0.90 ± 0.25	1.14 ± 0.48	0.67 ± 0.13	0.98 ± 0.22	0.87 ± 0.17
**pancreas**	0.68 ± 0.50	0.08 ± 0.03	0.30 ± 0.17	0.13 ± 0.06	0.26 ± 0.06	0.16 ± 0.14
**adrenal**	6.66 ± 2.33	0.26 ± 0.15**	2.89 ± 1.94	0.34 ± 0.09	2.14 ± 0.61	0.20 ± 0.04**
**kidney**	164.33 ± 50.20	1.62 ± 0.73**	73.86 ± 35.21	1.04 ± 0.14	83.22 ± 6.07	1.30 ± 0.25***
**lung**	1.67 ± 0.47	0.19 ± 0.09**	1.21 ± 0.48	0.39 ± 0.01	1.05 ± 0.14	0.43 ± 0.23*
**heart**	0.34 ± 0.08	0.09 ± 0.04**	0.31 ± 0.11	0.15 ± 0.00	0.22 ± 0.03	0.17 ± 0.07
**tumor**	**6.26** ± 0.82	0.18 ± 0.11***	**13.96** ± 5.20	0.41 ± 0.04*	**11.94** ± 2.29	0.37 ± 0.10***
**muscle**	0.28 ± 0.07	0.11 ± 0.08*	0.36 ± 0.18	0.15 ± 0.02	0.17 ± 0.02	0.10 ± 0.02*
**bone**	0.76 ± 0.57	0.56 ± 0.20	0.34 ± 0.14	0.17 ± 0.03	0.56 ± 0.14	0.57 ± 0.37
**brain**	0.05 ± 0.01	0.02 ± 0.01**	0.04 ± 0.01	0.02 ± 0.00	0.03 ± 0.01	0.03 ± 0.03
**T/M**	23.40 ± 5.00	1.91 ± 0.46***	49.67 ± 28.45	2.85 ± 0.70	72.20 ± 13.46	3.78 ± 0.17**
**T/B**	8.70 ± 1.74	0.75 ± 0.18***	17.12 ± 5.40	0.95 ± 0.10**	19.80 ± 7.23	0.72 ± 0.43*
**T/K**	0.04 ± 0.02	0.11 ± 0.03**	0.21 ± 0.08	0.41 ± 0.09	0.14 ± 0.02	0.29 ± 0.07*

Significance of differences between unblocked and blocked groups: $^{*}p < 0.05$; $^{**}p < 0.01$; $^{***}p < 0.001$.

Supplemental Table 3 – Complete biodistribution study for compounds **[^{18}F]7–8** and **[^{18}F]DCFPyL**

Tissue	**[^{18}F]7** 1h	1h blocked	**[^{18}F]8** 1h	1h blocked	**[^{18}F]DCFPyL** 1h

(%ID/g)	n =6	n = 4	n = 8	n = 4	n = 8
**blood**	0.13 ± 0.08	0.85 ± 1.37	0.56 ± 0.11	0.39 ± 0.07*	0.60 ± 0.13
**fat**	0.27 ± 0.14	0.02 ± 0.02	0.80 ± 0.28	0.06 ± 0.02***	1.05 ± 0.64
**testes**	0.18 ± 0.05	0.04 ± 0.01**	0.57 ± 0.12	0.18 ± 0.09***	0.57 ± 0.21
**intestine**	22.24 ± 2.79	26.68 ± 9.98	0.32 ± 0.06	0.26 ± 0.05	0.33 ± 0.07
**stomach**	0.21 ± 0.12	1.55 ± 2.10	0.11 ± 0.03	0.09 ± 0.04	0.12 ± 0.03
**spleen**	0.75 ± 0.36	0.15 ± 0.16	6.47 ± 2.17	0.12 ± 0.04***	3.98 ± 2.35
**liver**	0.83 ± 0.34	0.73 ± 0.21	0.20 ± 0.05	0.16 ± 0.04	1.82 ± 0.24
**pancreas**	0.13 ± 0.11	0.06 ± 0.06	0.46 ± 0.15	0.09 ± 0.03***	0.58 ± 0.32
**adrenal**	0.81 ± 0.25	0.06 ± 0.09**	7.72 ± 2.70	0.14 ± 0.03***	3.02 ± 2.14
**kidney**	20.35 ± 9.85	0.56 ± 0.18	143.85 ± 61.73	2.19 ± 0.44**	123.76 ± 37.67
**lung**	0.40 ± 0.13	0.12 ± 0.04*	1.97 ± 0.34	0.33 ± 0.06***	1.62 ± 0.68
**heart**	0.07 ± 0.02	0.04 ± 0.01	0.28 ± 0.07	0.13 ± 0.01**	0.35 ± 0.12
**tumor**	**5.09** ± 1.10	0.15 ± 0.06***	**16.66** ± 2.74	0.35 ± 0.03***	**11.64** ± 3.52
**muscle**	0.05 ± 0.01	0.24 ± 0.37	0.27 ± 0.06	0.13 ± 0.06**	0.29 ± 0.12
**bone**	0.10 ± 0.07	0.16 ± 0.25	0.25 ± 0.10	0.15 ± 0.02	0.33 ± 0.07
**brain**	0.01 ± 0.01	0.01 ± 0.01	0.02 ± 0.00	0.01 ± 0.00***	0.03 ± 0.01
**T/M**	117.13 ± 52.06	3.62 ± 3.62*	67.23 ± 25.93	3.07 ± 0.92***	43.67 ± 12.21
**T/B**	54.57 ± 38.49	1.56 ± 0.87	30.95 ± 7.76	0.92 ± 0.24***	19.64 ± 4.41
**T/K**	0.28 ± 0.22	0.28 ± 0.12	0.14 ± 0.07	0.17 ± 0.04	0.10 ± 0.02

Significance of differences between unblocked and blocked groups: $^{*}p < 0.05$; $^{**}p < 0.01$; $^{***}p < 0.001$.

IN VITRO PLASMA STABILITY STUDY

In vitro stability of **[^{18}F]1–8** and **[^{18}F]DCFPyL** was conducted in balb/c mouse plasma following previously published procedures (*1,2*), and monitored by radio-HPLC using the semi-preparative column eluted with various gradients of water/acetonitrile (0.1% TFA). No change in retention time was observed over the course of the study. Neither degradation nor release of free ^{18}F-fluoride was detected.

SYNTHESIS OF COLD PRECURSORS

Chemicals and instrumentation

Glu-ureido-Lys trifluoroacetate, *t*-butyl protected Glu-ureido-Lys (O*t*Bu-Glu(O*t*Bu)-ureido-Lys-O*t*Bu), methyl 4-[(dimethylamino)methyl]benzoate (**11**), 4-azidomethylbenzoic acid (**15**), 4-azidomethylnicotinic acid (**16**), *N*-propargyl-*N*,*N*-dimethylammoniomethyltrifluoroborate, *N*-propargylpyridinium *para*-trifluoroborate, DCFPyL and its fluorination precursor (*S*)-2-[3-[(*S*)-1-carboxy-5-(6-trimethylammonium-pyridine-3-carboxamido)pentyl]ureido]pentanedioic acid trifluoroacetate salt were prepared according to literature procedures (*1-7*). All other chemicals and solvents were obtained from commercial sources, and used without further purification. Purification and quality control of cold and radiolabeled PSMA-targeting peptidomimetics were performed on Agilent HPLC systems equipped with a model 1200 quaternary pump, a model 1200 UV absorbance detector (set at 220 nm), and a Bioscan (Washington, DC) NaI scintillation detector. The operation of Agilent HPLC systems was controlled using the Agilent ChemStation software. The HPLC columns used were a Phenomenex (Torrance, CA) Luna C_{18} semi-

preparative column (5 µ, 250 × 10 mm), a Phenomenex Luna C_{18} analytical column (5 µ, 250 × 4.6 mm), or a Phenomenex Jupiter C_{18} analytical column (10 µ, 250 × 4.6 mm). Lyophilization was conducted using a Labconco (Kansas City, MO) FreeZone 4.5 Plus freeze-drier. Mass analyses were performed using a Bruker (Billerica, MA) Esquire-LC/MS system with ESI ion source. Anion exchange columns were purchased from ORTG Inc. (Orkdale, TN), and C_{18} Sep-Pak cartridges (1 cm^3, 50 mg) were obtained from Waters (Milford, MA). ^{18}F-Fluoride was produced by the $^{18}O(p, n)^{18}F$ reaction using an Advanced Cyclotron Systems Inc. (Richmond, Canada) TR19 cyclotron. Radioactivity of ^{18}F-labeled tracers was measured using a Capintec (Ramsey, NJ) CRC®-25R/W dose calibrator, and the radioactivity of mouse tissues collected from biodistribution studies were counted using a Perkin Elmer (Waltham, MA) Wizard2 2480 automatic gamma counter.

Synthesis of precursors (Supplemental Supplemental Figure 2)

Compound **1** was prepared by coupling of the Glu-Lys ureido scaffold with a modified benzoic derivative: 4-[(dimethylamino)methyl]benzoate **11** was directly alkylated with (iodomethyl)boronic pinacol ester, which was then converted to the zwitterionic trifluoroborate. The coupling between the corresponding NHS ester **14** with deprotected Glu-ureido-Lys backbone (TFA salt) afforded **1**. Compounds **2**-**4** were prepared from azide-armed Glu-ureido-Lys scaffolds **19** and **20** (themselves prepared in similar fashion than **1**), onto which was attached the desired trifluoroborate ($AMBF_3$ or $pyrBF_3$) prosthetic *via* CuAAC. In a similar approach, the coupling of the desired prosthetic onto azide-armed PSMA-617 scaffolds (**22–24**, not shown, prepared on solid phase) afforded **5–8** (*see below*).

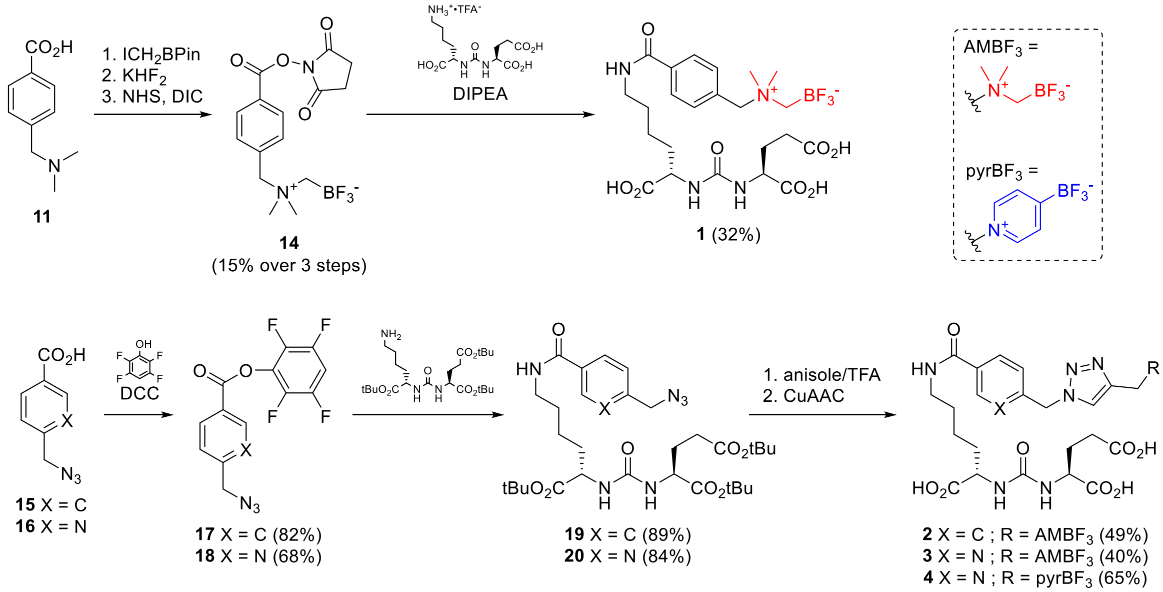

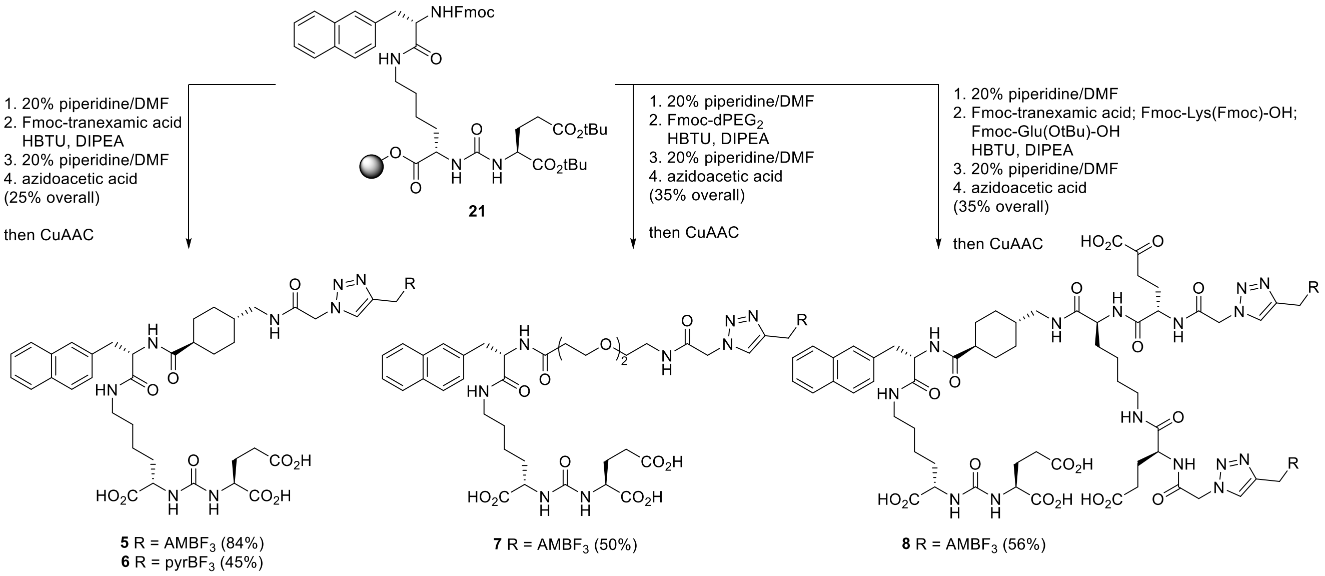

Supplemental Figure 2. General scheme for the synthesis of cold precursors 1–8.

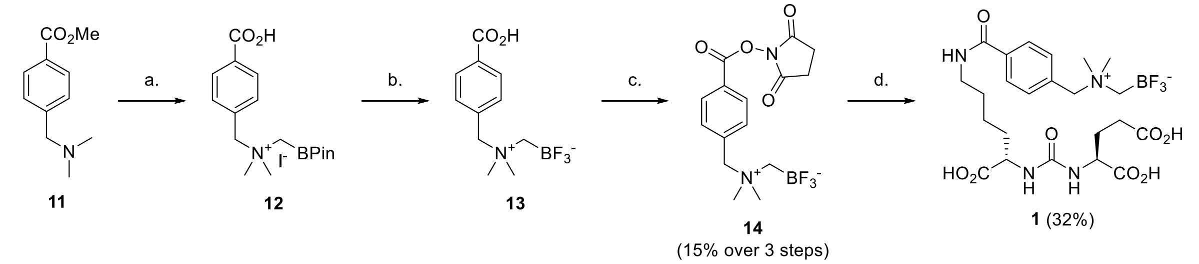

Conditions: a. (Iodomethyl)boronic pinacol ester (1.4 eq.), THF, rt, 24h; b. KHF_2 (6 eq.), HCl (23 eq.), MeOH/water, 60°C, 72 h; c. *N*-hydroxysuccinimide (1.05 eq.), *N*,*N'*-diisopropylcarbodiimide (1.05 eq.), DMF, rt, 24h; d. Glu-ureido-Lys trifluoroacetate (1.67 eq.), diisopropylethylamine (24.5 eq.), MeOH, 50°C, 72 h.

Synthesis of *N*-[4-(N-trifluoroborylmethyl-*N*,*N*-dimethylammoniomethyl)benzoyloxy]succinimide (14)

A solution of **11** (508 mg, 2.6 mmol) and (iodomethyl)boronic pinacol ester (1.0 g, 3.7 mmol) in distilled THF (10 mL) was stirred at room temperature for 24 h. The reaction mixture was concentrated under reduced pressure to obtain brown precipitant. The brown precipitant was washed with ether (10 mL × 5) and dried under vacuum. The crude intermediate **12** (1.4 g) and potassium hydrogen difluoride (1.2 g, 15.6 mmol) were dissolved in a mixture of H_2O (8 mL) and MeOH (10 mL) in a 50-mL plastic falcon tube. HCl (5 mL, 12 M) was added to the solution. The reaction mixture was heated at 60 °C for 3 days. After being cooled to room temperature, the reaction mixture was filtered through a short path of silica gel, eluted with acetonitrile (100 mL), and concentrated to give viscous oil (720 mg). The viscous oil containing **13** was dissolved in DMF (10 mL). *N*-Hydroxysuccinimide (317 mg, 2.75 mmol) was added, followed by *N*, *N*'-diisopropylcarbodiimide (348 mg, 2.76 mmol). The reaction mixture was stirred at room temperature for 24 h. The reaction mixture was then concentrated under reduced pressure and purified by HPLC using the semi-preparative column eluted with 25 % acetonitrile in H_2O at a flow rate of 4.5 mL/min and the retention time of the desired product was 10.6 min. The HPLC eluate

fractions containing the product were collected and lyophilized to yield compound **14** as white solid (150 mg, 15%). ^{1}H NMR (300 MHz, $CDCl_3$): δ 8.27 (d, *J* = 9.0 Hz 2H), δ 7.70 (d, *J* = 9.0 Hz, 2H), δ 4.56 (s, 2H), δ 3.06 (s, 6H), δ 2.95 (s, 4H), δ 2.57 (m, 2H). MS (ESI): calculated for $[M + Na]^+$ $C_{15}H_{18}BF_3N_2NaO_4$ 358.1; observed 381.1.

Synthesis of 1

Glu-ureido-Lys trifluoroacetate (38.8 mg, 0.122 mmol) and **14** (26 mg, 0.073 mmol) were dissolved in MeOH (3 mL) followed by *N*,*N*-diisopropylethylamine (312 µL, 1.792 mmol). The reaction mixture was heated at 50 °C and stirred for 3 days and then concentrated under reduced pressure. The product was purified by HPLC using the semi-preparative column eluted with 15-35 % acetonitrile (0.5% acetic acid) in H_2O (0.5% acetic acid) at a flow rate of 4.5 mL/min. The HPLC eluate fractions containing the product were collected and lyophilized to yield **1** as a white solid (13 mg, 32%). ^{1}H NMR (300 MHz, D_2O): δ 7.77 (d, *J* = 9 Hz, 2H), δ 7.60 (d, *J* = 9 Hz, 2H), δ 4.42 (s, 2H), δ 4.15 (m, 3H), δ 3.36 (t, *J* = 6.0, 2H), δ 2.95 (s, 6H), δ 2.41 (t, *J* = 6.0 Hz, 2H), δ 2.13-2.02 (m, 2H), δ 1.91-1.75 (m, 2H), δ 1.71-1.55 (m, 3H), δ 1.50-1.32 (m, 2H). MS (ESI): calculated for $[M + H]^+$ $C_{23}H_{35}BF_3N_4O_8$ = 563.3; observed 563.4.

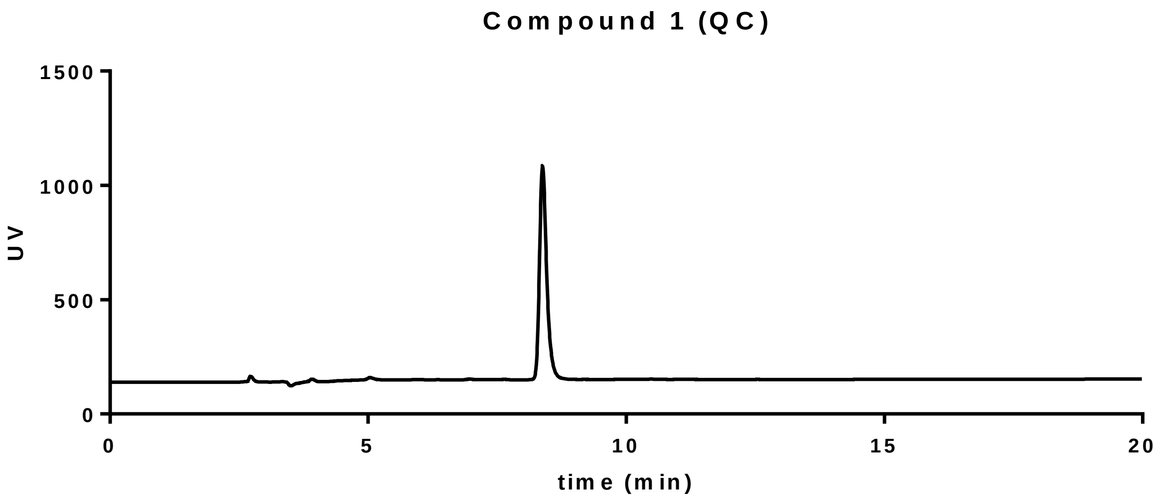

Supplemental Figure 3 - HPLC trace of pure **1**.

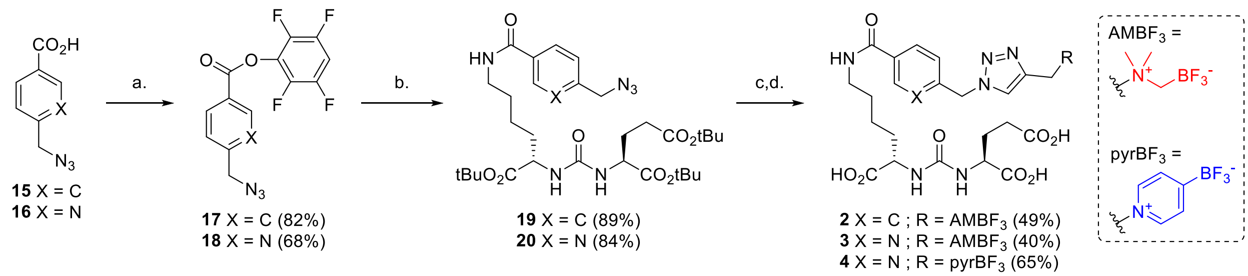

Conditions: a. 2,3,5,6-tetrafluorophenol (1.1 to 1.5 eq.), *N*,*N*'-dicyclohexylcarbodiimide (0.9 to 1.5 eq.), CH_2Cl_2, 0°C, 3 h; b. *t*-butyl protected Glu-ureido-Lys (0.67 to 0.83 eq.), THF, rt, 16 h; c. 3% anisole in TFA, rt, 4 h; d. For **2** and **3**: *N*-propargyl-*N*,*N*-dimethyl-ammoniomethyltrifluoroborate (3 eq.), $CuSO_4$ (3 eq.), Na ascorbate (6 eq.), MeCN/water, 45°C, 2 h; For **4**: *N*-propargylpyridinium

para-trifluoroborate (0.4 eq., limiting reagent), $CuSO_4$ (0.18 eq.), Na ascorbate (0.36 eq.), $NaHCO_3$ (4 eq.), DMF/water, rt, 2 h.

Synthesis of 2,3,5,6-tetrafluorophenyl 4-azidomethylbenzoate (17)

A solution of 4-(azidomethyl)benzoic acid **15** (719 mg, 4.0 mmol) and 2,3,5,6-tetrafluorophenol (731 mg, 4.4 mmol) in CH_2Cl_2 (20 mL) was cooled in an ice/water bath. *N*,*N*'-dicyclohexylcarbodiimide (743 mg, 3.6 mmol) was added to the reaction mixture and stirred for 3 h. The reaction mixture was filtered and the filtrate was evaporated. After evaporation, the residue was dissolved in hexane (100 mL), and the solution was filtered again and washed with 1N NaOH aqueous solution (100 mL). The organic phase was dried over anhydrous magnesium sulfate, concentrated under reduced pressure, and purified by chromatography on silica gel eluted with 1:5 ether/hexane to obtain the desired product **17** as white solid (1.06 g, 82%). ^1H NMR (300 MHz, $CDCl_3$): δ 8.25 (d, *J* = 9 Hz, 2H), δ 7.52 (d, *J* = 9 Hz, 2H), δ 7.06 (m, 1H), δ 4.42 (s, 2H), δ 4.15 (m, *J* = 4.9, 2H), δ 3.36 (t, *J* = 6.0 Hz, 2H), δ 2.95 (s, 6H), δ 2.41 (t, *J* = 6.0 Hz, 2H), δ 4.50 (s, 2H). MS (ESI): calculated for $[M]^-$ $C_{14}H_7F_4N_3O_2$ 325.1; observed 325.6.

Synthesis of (*S*)-2-[3-[5-(4-azidomethylbenzoylamino)-(*S*)-1-(*tert*-butoxyloxycarbonyl)pentyl]ureido] pentanedioic acid bis(4-*tert*-butyl) ester (19)

A solution of *t*-butyl protected Glu-ureido-Lys (101.9 mg, 0.21 mmol) and **17** (100.1 mg, 0.31 mmol) in THF (20 mL) was stirred overnight at room temperature. The reaction mixture was concentrated under reduced pressure and purified by chromatography on silica gel eluted with 1:1 hexane/EtOAc to obtain the desired product **19** as a light-yellow oil (120.6 mg, 89%). ^1H NMR (300 MHz, $CDCl_3$): δ 7.89 (d, *J* = 8.2 Hz 2H), δ 7.37 (d, *J* = 8.2 Hz, 2H), δ 7.05 (bt, 1H), δ 5.43 (m, 1H), δ 5.33 (m, 1H), δ 4.39 (s, 2H), δ 4.25 (m, 2H), δ 3.53-3.36 (m, 2H), δ 2.28 (m, 2H), δ 2.10-1.96 (m,1H), δ 1.87-1.75 (m, 2H), δ 1.69-1.56 (m, 3H), δ 1.43 (s, 18H), δ 1.40 (s,9H). MS (ESI): calculated for $[M + H]^+$ $C_{32}H_{51}N_6O_8$ 647.4; observed 647.6.

Synthesis of 2

A solution of **19** (98 mg, 0.15 mmol) in TFA (5 mL) containing 3% anisole was stirred at room temperature. After 4 h, the reaction mixture was concentrated under reduced pressure. The residue was dissolved in water (1 mL) and wash with hexane (1 mL × 3) to remove anisole. The aqueous phase was lyophilized to obtain a yellow oil. The crude product was purified by HPLC using the semi-preparative column eluted with 25-50 % acetonitrile (0.1% TFA) in water (0.1% TFA) in 25 min at a flow rate of 4.5 mL/min, and the retention time of the desired product was 10 min. The HPLC eluate fractions containing the product were collected and lyophilized to yield **deprotected 19** as white solid (71 mg, 99%). ^1H NMR (300 MHz, D_2O): δ 7.72 (d, *J* = 8.2 Hz 2H), δ 7.47 (d, *J* = 8.2 Hz, 2H), δ 4.65-4.90 (m, 2H), δ 4.46 (s, 2H), δ 4.16 (dd, *J* = 4.9, 8.8 Hz, 2H), δ 3.37 (t, *J* = 6.8 Hz, 2H), δ 2.43 (t, *J* = 7.4 Hz, 2H), δ 2.10-2.15 (m,1H), δ 1.75-1.60 (m, 3H), δ 1.47-1.43 (m, 2H). MS (ESI): calculated for $[M + H]^+$ $C_{20}H_{27}N_6O_8$ 479.2; observed 479.3.

A solution of **deprotected 19** (10.5 mg, 0.022 mmol), *N*-propargyl-*N*,*N*-dimethyl-ammoniomethyltrifluoroborate (10.7 mg, 0.065 mmol), 1 M $CuSO_4$ (65 μL), and 1 M sodium ascorbate (162.5 μL) in acetonitrile (150 μL) was incubated at 45 °C for 2 h. The reaction mixture was purified by HPLC using the semi-preparative column eluted with 15-35 % acetonitrile (0.5 % acetic acid) in water (0.5 % acetic acid) at a flow rate of 4.5 mL/min. The HPLC eluate fractions containing the product were collected and lyophilized to yield **2** as white solid (7 mg, 49 %). ^1H

NMR (300 MHz, D_2O): δ 8.31 (s, 1H), δ 7.69 (d, *J* = 9 Hz, 2H), δ 7.38 (d, *J* = 9 Hz, 2H), δ 5.69 (s, 2H), δ 4.72 (s, 2H), δ 4.03 (m, 2H), δ 3.33 (m, 2H), δ 3.13 (m, 1H), δ 2.97 (s, 6H), δ 2.40-2.32 (m, 3H), δ 1.99 (m, 2H), δ 1.88-1.69 (m, 2H), δ 1.67-1.50 (m, 2H), δ 1.45-1.30 (m, 2H). MS (ESI): calculated for $[M + H]^+$ $C_{26}H_{38}BF_3N_7O_8$ 644.3; observed 644.4

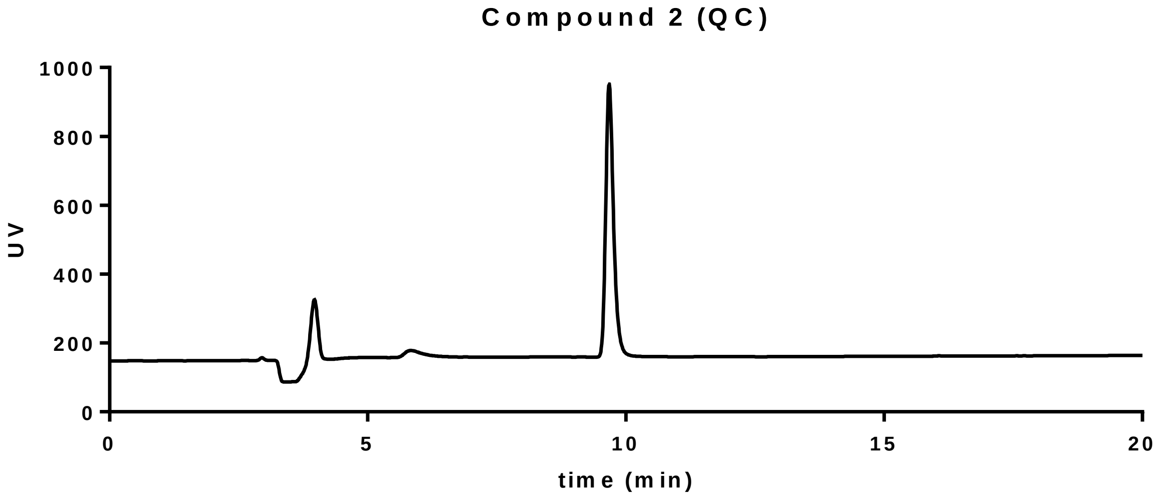

Supplemental Figure 4 - HPLC trace of pure **2**.

Synthesis of 2,3,5,6-tetrafluorophenyl 4-azidomethylnicotinate (18)

A solution of 6-(azidomethyl)nicotinic acid **16** (507 mg, 2.8 mmol) and 2,3,5,6-tetrafluorophenol (700 mg, 4.2 mmol) in CH_2Cl_2 (20 mL) was cooled in an ice/water bath. *N*,*N*'-dicyclohexylcarbodiimide (865 mg, 4.2 mmol) was added to the reaction mixture and stirred for 3 h. The reaction mixture was filtered and the filtrate was concentrated under reduced pressure, and purified by chromatography on silica gel eluted with 1:30 ether/hexane to obtain the desired product **2** as white solid (626.7 mg, 68%). ^1H NMR (300 MHz, $CDCl_3$): δ 9.36 (d, *J*= 2.2 Hz, 1H), δ 8.49 (dd, *J*= 8.0, 2.2 Hz, 1H), δ 7.57 (d, *J*= 8.0 Hz, 1H), δ 7.08 (m, 1H), δ 4.64 (s, 2H) MS (ESI): calculated for $C_{13}H_6F_4N_4O_2$ $[M + H]^+$= 327.05; observed $[M + H]^+$= 327.30.

Synthesis of (*S*)-2-[3-[5-(4-azidomethylpicolylamino)-(*S*)-1-(*tert*-butoxyloxycarbonyl)pentyl]ureido] pentanedioic acid bis(4-*tert*-butyl) ester (20)

A solution of *t*-butyl protected Glu-ureido-Lys (141.1 mg, 0.30 mmol) and **18** (118.0 mg, 0.36 mmol) in THF (20 mL) was stirred overnight at room temperature. The reaction mixture was concentrated under reduced pressure and purified by chromatography on silica gel eluted with 2:3 hexane/EtOAc to obtain the desired product **20** as light yellow oil (163.2 mg, 84%). ^1H NMR (300 MHz, $CDCl_3$): δ 9.09 (d, *J*= 1.9 Hz 1H), δ 8.26 (dd, *J*= 8.3, 2.2 Hz 1H), δ 7.45 (bt, 1H), δ 7.43 (d, *J*= 8.3 Hz, 1H), δ 5.50 (d, *J*= 7.7 Hz 1H), δ 5.32 (d, *J*= 8.0 Hz 1H), δ 4.53 (s, 2H), δ 4.23 (m, 2H), δ 3.57-3.38 (m, 2H), δ 2.29 (m, 2H), δ 2.20-1.97 (m,1H), δ 1.82-1.76 (m, 2H), δ 1.68-1.56 (m, 3H), δ 1.43 (s, 18H), δ 1.38 (s,9H). MS (ESI): calculated for $C_{31}H_{49}N_7O_8$ $[M + H]^+$= 648.37; observed $[M + H]^+$= 648.60.

Synthesis of 3

A solution of **20** (163.2 mg, 0.15 mmol) in TFA (5 mL) containing 3% anisole was stirred at room temperature. After 4 h, the reaction mixture was concentrated under reduced pressure. The residue was dissolved in water (2 mL) and wash with hexane (2 mL × 3) to remove anisole. The aqueous phase was lyophilized to obtain crude a yellow oil (180.2 mg). The crude product (20.0 mg, 0.04 mmol), *N*-propargyl-*N*,*N*-dimethyl-ammoniomethyltrifluoroborate (20.6 mg, 0.13 mmol), 1 M $CuSO_4$ (124 µL), and 1 M sodium ascorbate (310 µL) in acetonitrile (150 µL) and 5 % NH_4OH (300 µL) was incubated at 45 °C for 2 h. The reaction mixture was purified by HPLC using semi-preparative column eluted with 3-13 % acetonitrile in ammonium formate buffer (40 mM, pH 6.0) at a flow rate of 4.5 mL/min. **3** was obtained as white solid (10.4 mg, 40 %). MS (ESI): calculated for $C_{25}H_{36}BF_3N_8O_8$ $[M + H]^+$= 645.28; observed $[M + H]^+$= 645.50.

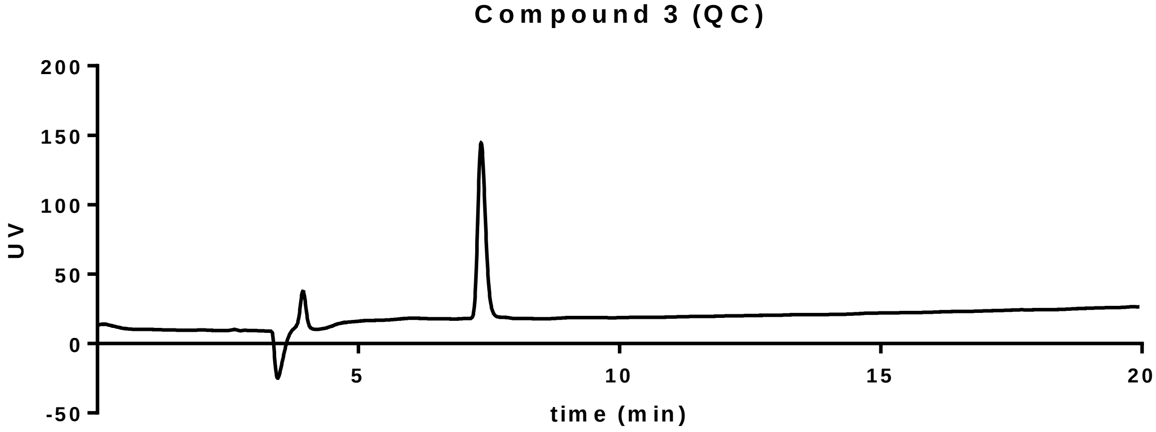

Supplemental Figure 5 - HPLC trace of pure **3**.

Synthesis of 4

To a solution of *N*-propargylpyridinium *para*-trifluoroborate (1 eq., 2.6 mg, 14 µmol) and deprotected **20** (2.5 eq., 16.8 mg, 35 µmol) in DMF (500 µL) at room temperature was added a bright yellow solution of $Cu^{(I)}$ prepared by mixing 0.1M aq. $CuSO_4$ (10 mol%, 14 µL, 1.4 µmol), 0.2M aq. sodium ascorbate (20 mol%, 14 µL, 2.8 µmol) and 1M aq. sodium bicarbonate (1 eq., 14 µL, 14 µmol) with H_2O (58 µL). The mixture was stirred at room temperature for 2h, but low conversion was assessed by TLC. An excess of 1M aq. sodium bicarbonate (10 eq., 141 µL, 141 µmol) was added, causing a gas release. To ensure reaction rate, another portion of 0.1 M aq. $CuSO_4$ (35 mol%, 49 µL, 4.9 µmol) and 0.2M aq. sodium ascorbate (70 mol%, 49 µL, 98 µmol) were added. The mixture was stirred at room temperature for 5 min. The reaction was then quenched with 10 drops of ammonia and then filtered through a small silica gel pad (2 cm high, 0.5 cm) built in a Pasteur pipet, eluting with a 9.5/9.5/1 mixture of MeCN/MeOH/ammonium hydroxide (10 mL). The filtrate was concentrated, then diluted with water (4 mL), frozen and lyophilized. The dry residue was purified by HPLC using semi-preparative column eluted with 0-30 % acetonitrile (0.1% formic acid) in water 0.1% formic acid) at a flow rate of 2 mL/min (retention time = 19.0 min) to afford pure **4** (6.1 mg, 65% yield). ESI-HRMS (TOF) *m/z* $[M-H]^-$ 662.2352; calc. 662.2346 for $C_{27}H_{31}N_8O_8{}^{10}BF_3$.

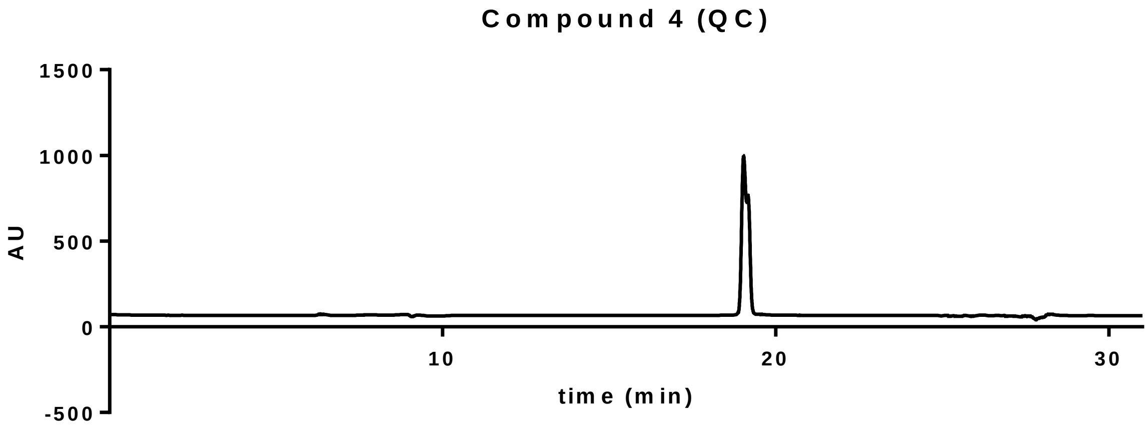

Supplemental Figure 6 - HPLC trace of pure **4**.

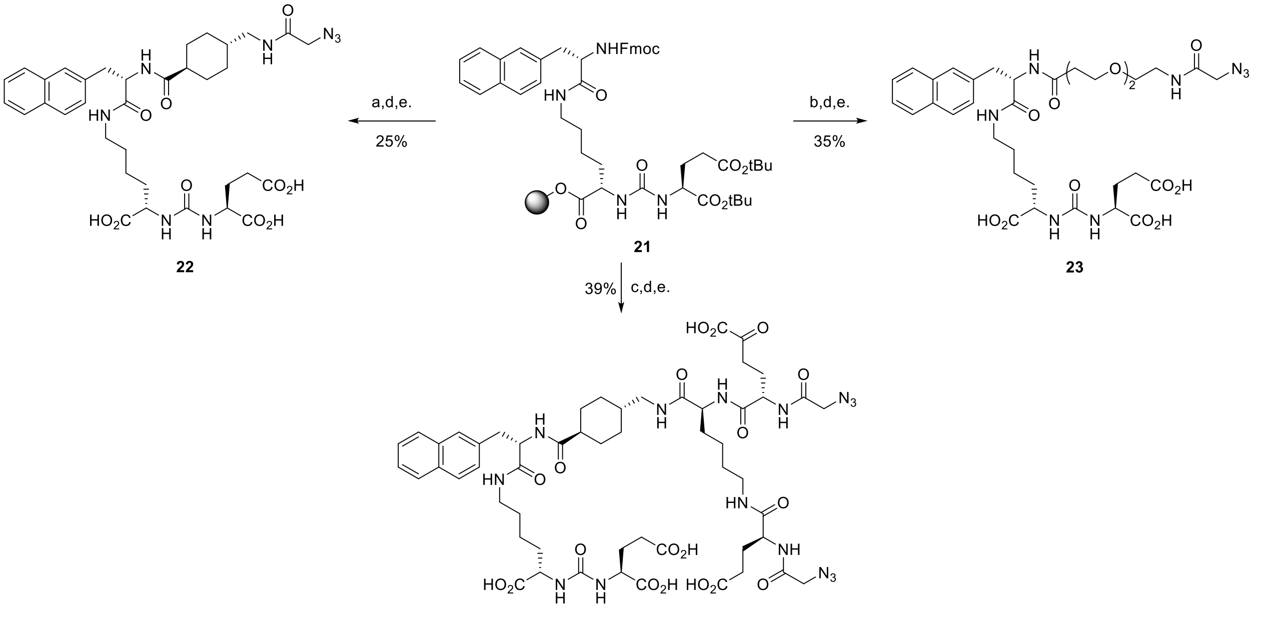

Conditions: a. (i) 20% piperidine/DMF (v/v), rt, 30 min; (ii) Fmoc-tranexamic acid, HBTU, DIPEA, rt, 2 h; b. (i) 20% piperidine/DMF (v/v), rt, 30 min; (ii) Fmoc-dPEG2, HBTU, DIPEA, rt, 2 h; c. (i) 20% piperidine/DMF (v/v), rt, 30 min; (ii) Fmoc-dPEG2; Fmoc-Lys(Fmoc)-OH; Fmoc-Glu(OtBu)-OH, HBTU, DIPEA, rt, 2 h; d. (i) 20% piperidine/DMF (v/v), rt, 30 min; (ii) azidoacetic acid (5 eq.), DCC (5 eq.), NHS (6 eq.), rt, 2 h; e. TFA/TIS 95:5 (v/v), rt, 2 h.

Synthesis of 21

Resin-bound **21** was synthesized on solid phase by following reported procedures.(*8*)

Synthesis of 22

After Fmoc deprotection of **21**, Fmoc-protected tranexamic acid was coupled to the *N*-terminus according to a reported procedure.(*8*) After Fmoc deprotection, 2-azidoacetic acid (5 equivalents)

was coupled to the *N*-terminus using the *in situ* activating reagent *N,N'*-diisopropylcarbodiimide (5 eq.) and *N*-hydroxysuccinimide (6 eq.) in DMF for 2 h at room temperature. At the end, the peptide was deprotected and simultaneously cleaved from the resin by treating the beads with a TFA/TIS 95:5 (v/v) mixture for 2 h at room temperature. After filtration, the peptide was precipitated by the addition of cold diethyl ether to the TFA solution. The crude peptide was purified by HPLC using a semi-preparative column eluted with 35-45 % acetonitrile (0.1% TFA) in water (0.1% TFA) at a flow rate of 4.5 mL/min. Collection of the peak with a retention time of 9.1 min afforded **22** in 25 % yield. MS (ESI): calculated for $C_{35}H_{46}N_8O_{10}$ $[M + H]^+$= 739.80; observed $[M + H]^+$= 740.26.

Synthesis of 23

After Fmoc deprotection of **21**, Fmoc-protected $dPEG_2$ acid was coupled to the *N*-terminus using standard solid-phase peptide synthesis. The Fmoc protecting group was removed and 2-azidoacetic acid (5 equivalents) was coupled to the *N*-terminus with the *in situ* activating reagent *N,N'*-diisopropylcarbodiimide (5 equivalents) and *N*-hydroxysuccinimide (6 equivalents) in DMF for 2 h at room temperature. At the end, the peptide was deprotected and simultaneously cleaved from the resin by treating with 95/5 TFA/TIS for 2 h at room temperature. After filtration, the peptide was precipitated by the addition of cold diethyl ether to the TFA solution. The crude peptide was purified by HPLC using the semi-preparative column eluted with 31-40 % acetonitrile (0.1% TFA) in water at a flow rate of 4.5 mL/min. The retention time was 9.8 min, and the yield of the peptide **23** was 35.5 %. MS (ESI): calculated for $C_{34}H_{46}N_8O_{12}$ $[M + H]^+$= 759.33; observed $[M + H]^+$= 759.50.

Synthesis of 24

After Fmoc deprotection of **21**, Fmoc-protected tranexamic acid was coupled to the *N*-terminus followed by Fmoc-Lys(Fmoc)-OH and Fmoc-Glu(OtBu)-OH via solid-phase peptide synthesis using Fmoc-based chemistry. After Fmoc deprotection, 2-azidoacetic acid (5 equivalents) was coupled to the *N*-terminus using the *in situ* activating reagent *N,N'*-diisopropylcarbodiimide (5 eq.) and *N*-hydroxysuccinimide (6 eq.) in DMF for 2 h at room temperature. At the end, the peptide was deprotected and simultaneously cleaved from the resin by treating the beads with a TFA/TIS 95:5 (v/v) mixture for 2 h at room temperature. After filtration, the peptide was precipitated by the addition of cold diethyl ether to the TFA solution. The crude peptide was purified by HPLC using a semi-preparative column eluted with 33 % acetonitrile (0.1% TFA) in water (0.1% TFA) at a flow rate of 4.5 mL/min. Collection of the peak with a retention time of 10.1 min afforded **22** in 39 % yield. MS (ESI): calculated for $C_{53}H_{73}N_{15}O_{18}$ $[M + H]^+$= 1208.53; observed $[M + H]^+$= 1208.68.

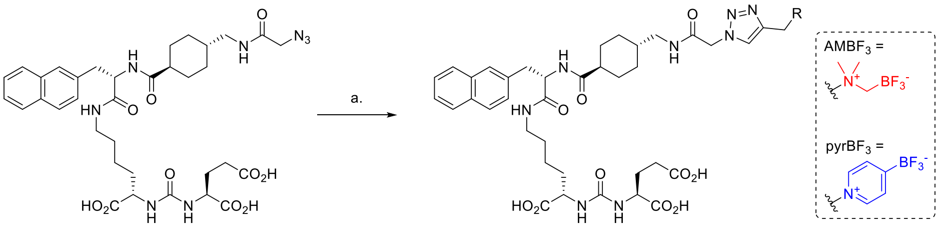

22

5 R = $AMBF_3$ (84%)
6 R = $pyrBF_3$ (45%)

Conditions: a. $AMBF_3$ or $pyrBF_3$ (2–5 eq.), $CuSO_4$ (cat.), Na ascorbate (cat.), NH_4OH, MeCN/H_2O, 45°C, 2 h.

Synthesis of 5

A solution of **22** (3.8 mg, 5 µmol), *N*-propargyl-*N*,*N*-dimethyl-ammoniomethyltrifluoroborate (4 mg, 24.2 µmol), 1 M $CuSO_4$ (25 µL), and 1 M sodium ascorbate (70 µL) in acetonitrile (150 µL) and 5 % NH_4OH (150 µL) was incubated at 45 °C oil bath for 2 h. The reaction mixture was purified by HPLC using the semi-preparative column eluted with 21 % acetonitrile and 79 % ammonia formate buffer (40 mM, pH 6.0) at a flow rate of 4.5 mL/min. The yield of the peptide was 84 %. MS (ESI): calculated for $C_{41}H_{57}BF_3N_9O_{10}$ $[M + H]^+$= 904.44; observed $[M + H]^+$= 904.60.

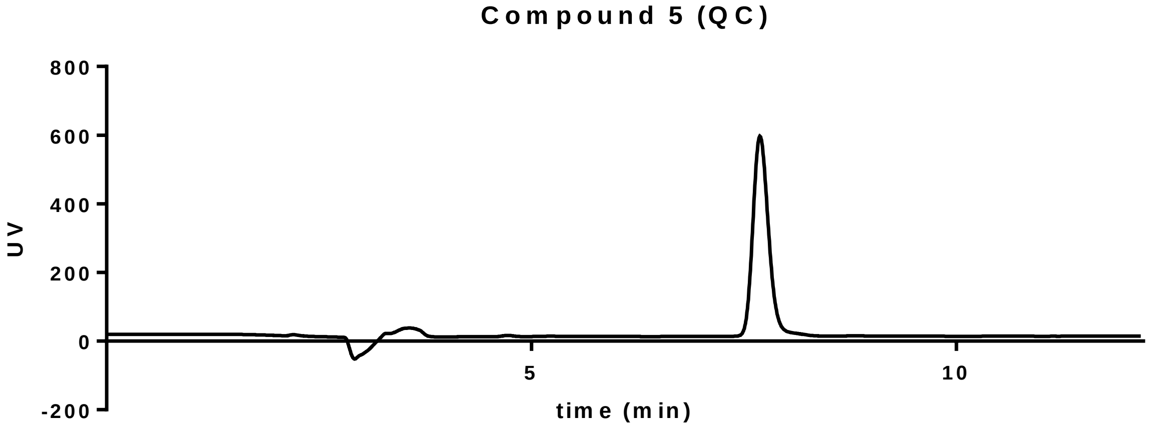

Supplemental Figure 7 - HPLC trace of pure **5**.

Synthesis of 6

A solution of **22** (2.5 mg, 3.4 µmol), *N*-propargyl-*para*-pyridiniumtrifluoroborate (1.3 mg, 6.8 µmol), 1 M $CuSO_4$ (25 µL), and 1 M sodium ascorbate (70 µL) in acetonitrile (150 µL) and 5 % NH_4OH (150 µL) was incubated at 45 °C oil bath for 2 h. The reaction mixture was purified by HPLC using the semi-preparative column eluted with a gradient of acetonitrile and formate buffer (40 mM, pH 6.0) at a flow rate of 2 mL/min to afford the peptide with 45 % yield. ESI-HRMS (TOF) *m/z* $[M-H]^-$ 921.3918; calc. 921.3919 for $C_{43}H_{52}BF_3N_9O_{10}$.

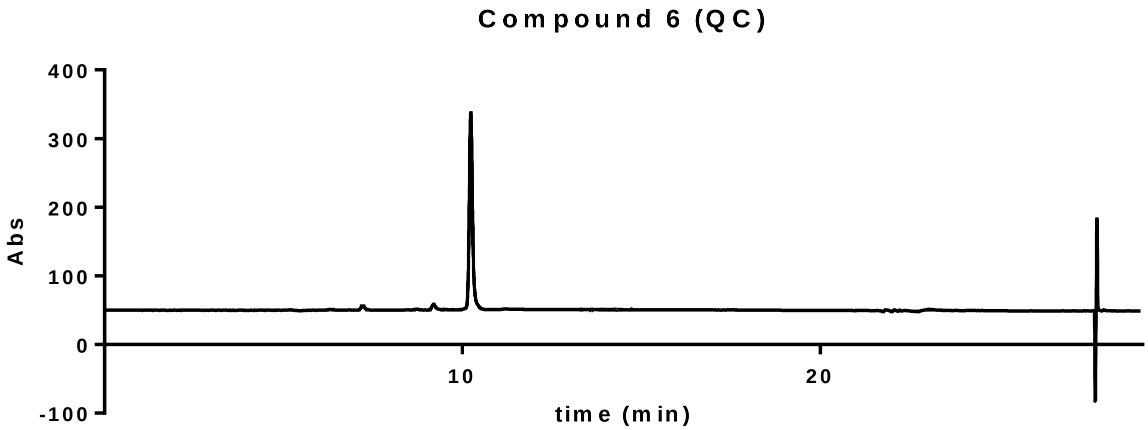

Supplemental Figure 8 - HPLC trace of pure **6**.

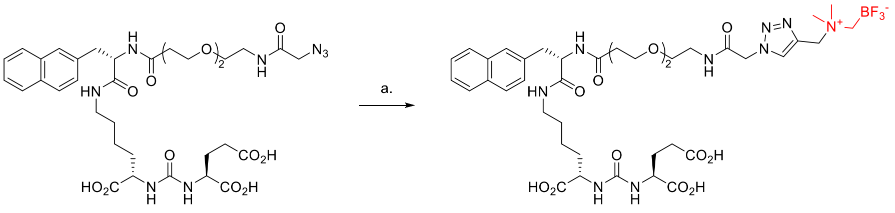

23 **7** (50%)

Conditions: a. $AMBF_3$ or $pyrBF_3$ (3.5 eq.), $CuSO_4$ (cat.), Na ascorbate (cat.), NH_4OH, MeCN/H_2O, 45°C, 2 h.

Synthesis of 7

A solution of **23** (10.5 mg, 0.014 mmol), *N*-propargyl-*N*,*N*-dimethyl-ammoniomethyltrifluoroborate (8.0 mg, 48.6 μmol), 1 M $CuSO_4$ (30 μL), and 1 M sodium ascorbate (72 μL) in acetonitrile (100 μL) and 5 % NH_4OH (100 μL) was incubated at 45 °C oil bath for 2 h. The reaction mixture was purified by HPLC using the semi-preparative column eluted with 20 % acetonitrile and 80 % ammonia formate buffer (40 mM, pH 6.0) at a flow rate of 4.5 mL/min. The yield of the peptide was 50.0 %. MS (ESI): calculated for $C_{40}H_{57}BF_3N_9O_{12}$ $[M + Na]^+$= 946.41; observed $[M + Na]^+$= 946.60.

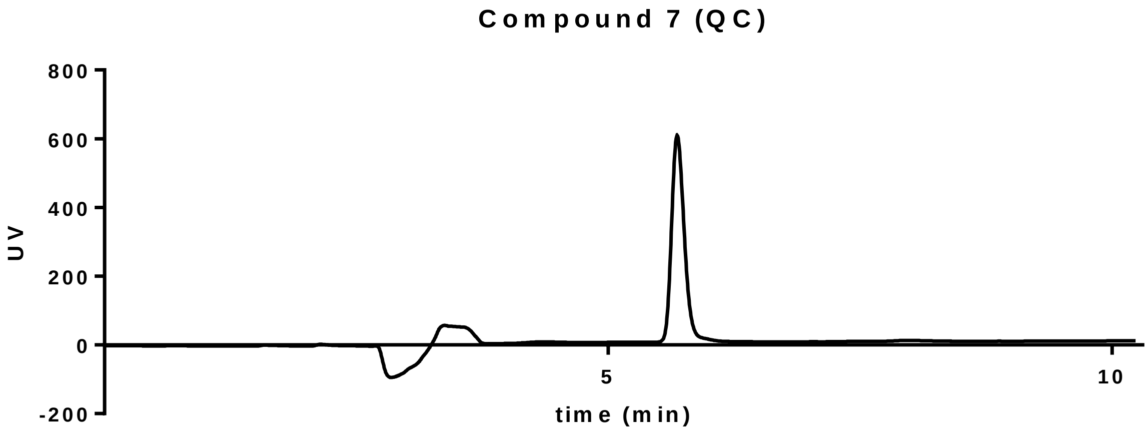

Supplemental Figure 9 - HPLC trace of pure **7**.

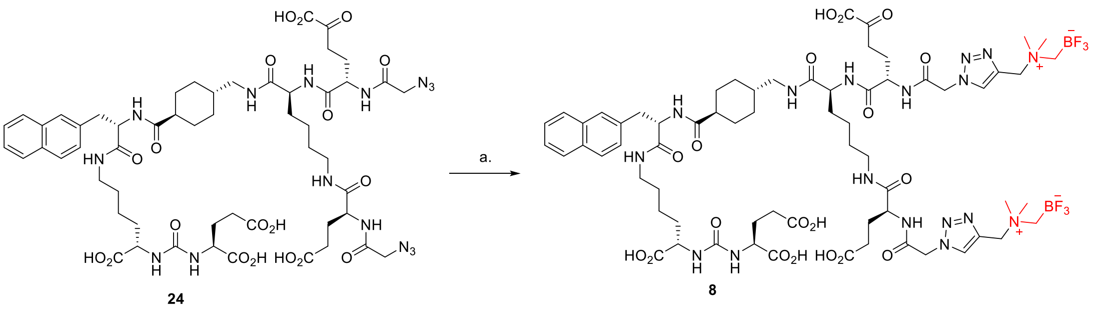

Conditions: a. $AMBF_3$ (6 eq.), $CuSO_4$ (cat.), Na ascorbate (cat.), NH_4OH, MeCN/H_2O, 45°C, 2 h.

Synthesis of 8

A solution of **24** (6.0 mg, 5.0 μmol), *N*-propargyl-*N*,*N*-dimethyl-ammoniomethyltrifluoroborate (4.9 mg, 30.0 μmol), 1 M $CuSO_4$ (37.5 μL), and 1 M sodium ascorbate (94 μL) in acetonitrile (150 μL) and 5 % NH_4OH (150 μL) was incubated at 45 °C oil bath for 2 h. The reaction mixture was purified by HPLC using the semi-preparative column eluted with 15 % acetonitrile and 85 % ammonia formate buffer (40 mM, pH 6.0) at a flow rate of 4.5 mL/min. The yield of the peptide was 56.0 %. MS (ESI): calculated for $C_{65}H_{95}B_2F_6N_{17}O_{18}$ $[M + H]^+$= 1538.72; observed $[M + H]^+$= 1538.88.

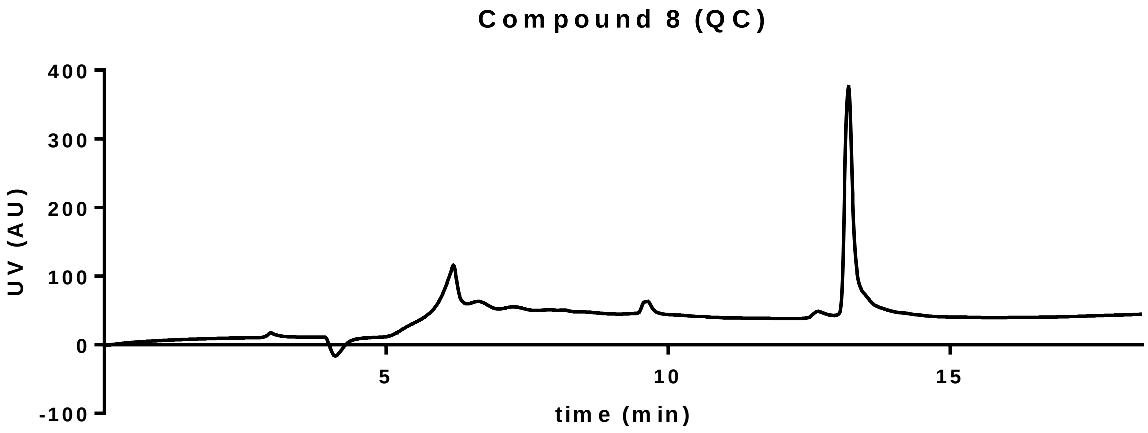

Supplemental Figure 10 – HPLC trace of pure **8**.

QC ANALYSIS OF TRACERS [^{18}F]1–8

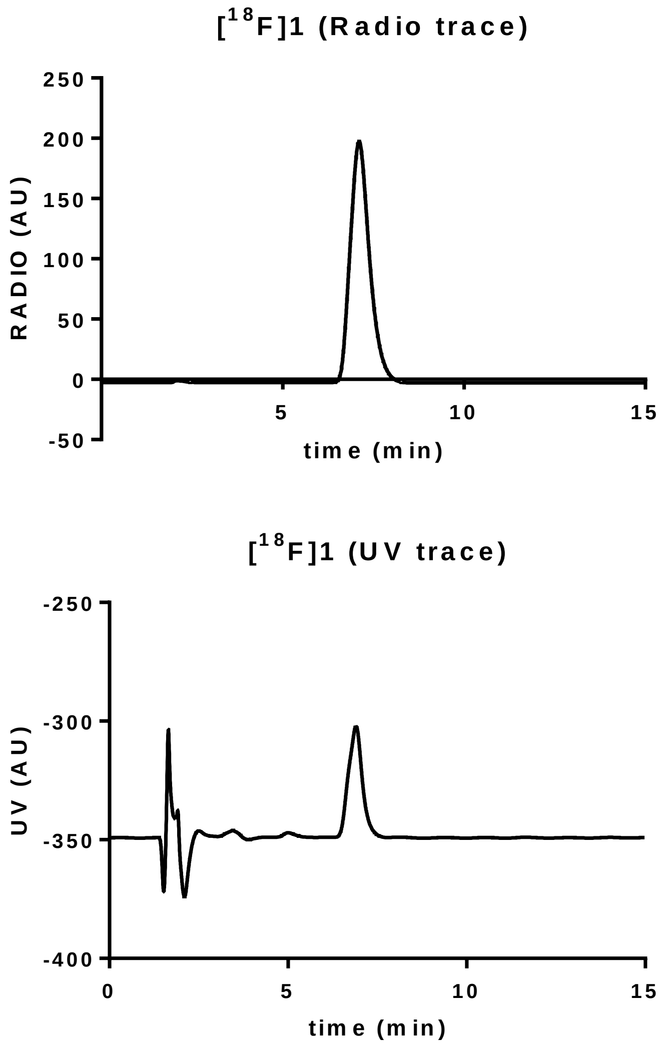

Supplemental Figure 11 - QC analysis of **[^{18}F]1**.

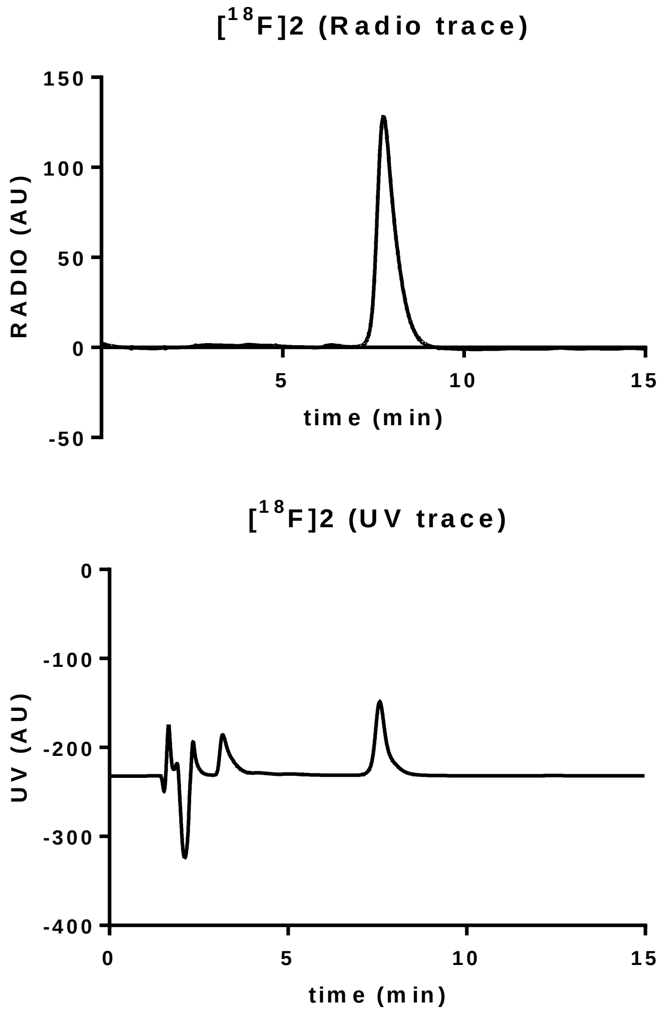

Supplemental Figure 12 - QC analysis of **[^{18}F]2**.

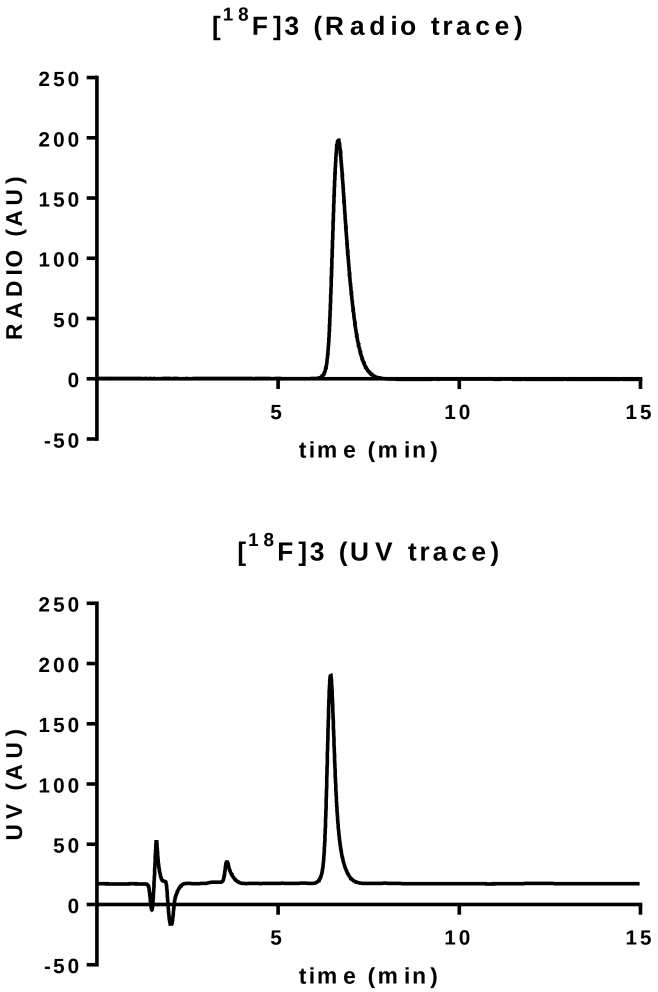

Supplemental Figure 13 - QC analysis of **[18F]3**.

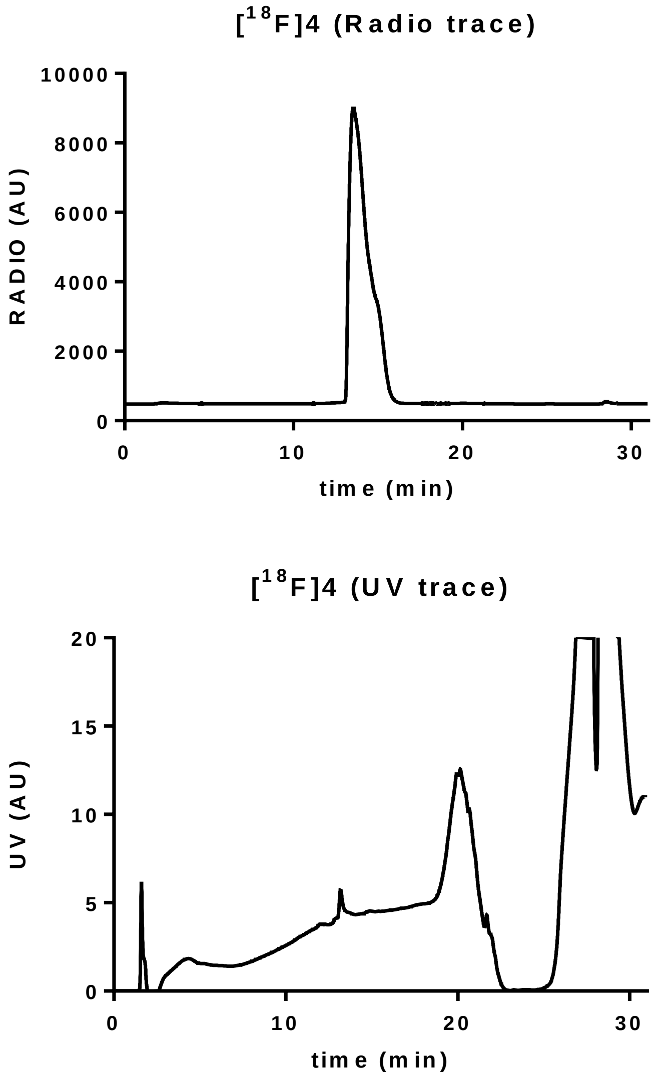

Supplemental Figure 14 - QC analysis of **[^{18}F]4**.

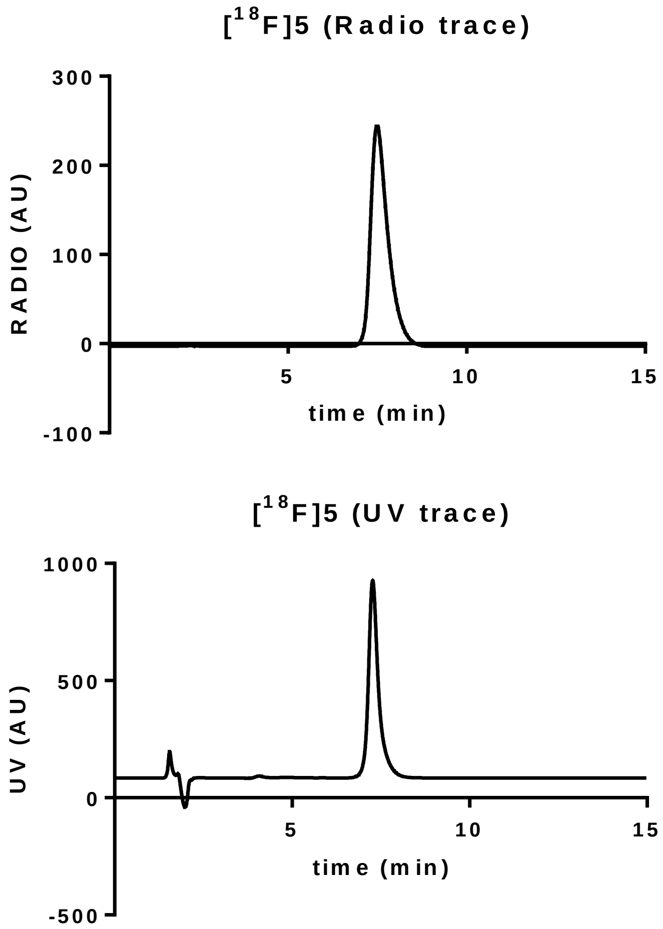

Supplemental Figure 15 - QC analysis of **[^{18}F]5**.

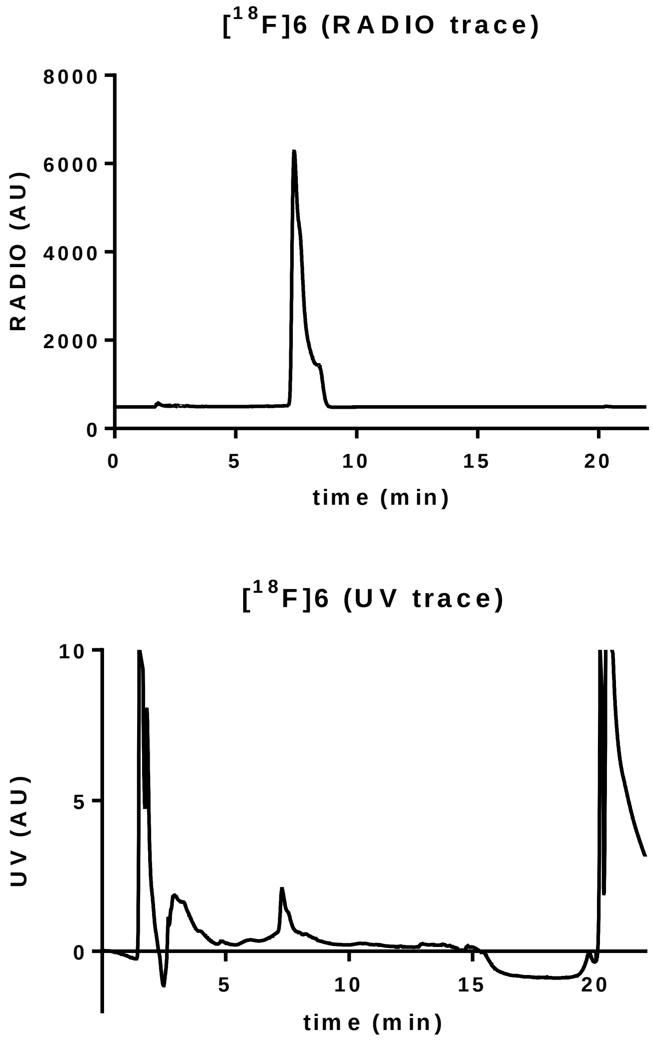

Supplemental Figure 16 - QC analysis of **[^{18}F]6**.

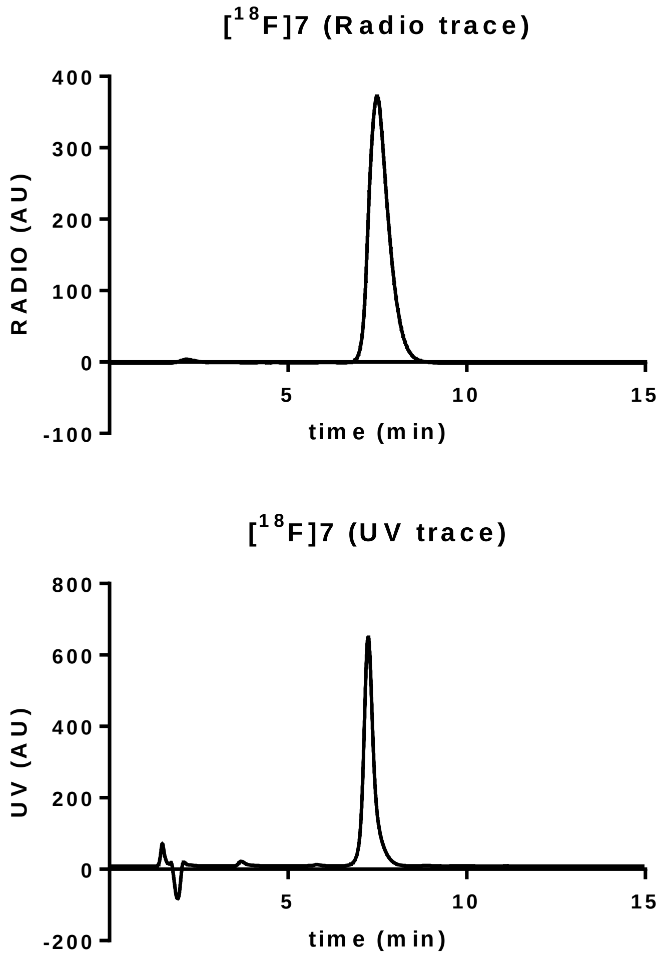

Supplemental Figure 17 - QC analysis of **[^{18}F]7**.

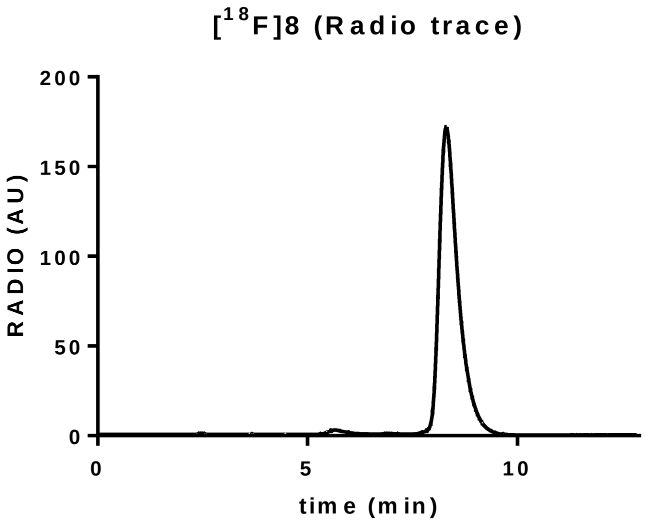

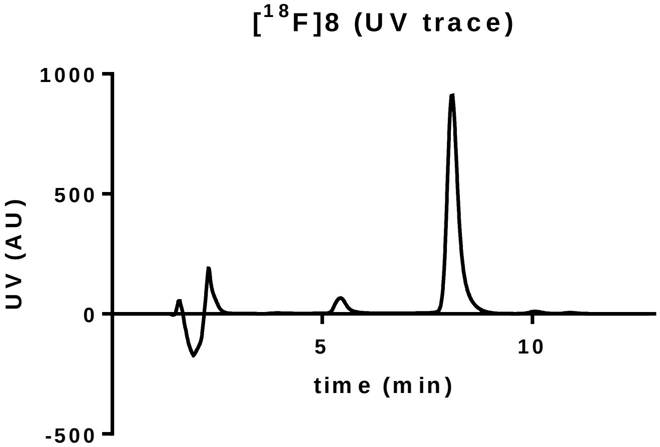

Supplemental Figure 18 - QC analysis of **[^{18}F]8**.

1. Liu ZB, Pourghiasian M, Benard F, Pan JH, Lin KS, Perrin DM. Preclinical Evaluation of a High-Affinity F-18-Trifluoroborate Octreotate Derivative for Somatostatin Receptor Imaging. *Journal of Nuclear Medicine.* 2014;55:1499-1505.

2. Pourghiasian M, Liu ZB, Pan JH, et al. F-18-AmBF3-MJ9: A novel radiofluorinated bombesin derivative for prostate cancer imaging. *Bioorganic & Medicinal Chemistry.* 2015;23:1500-1506.

3. Maresca KP, Hillier SM, Femia FJ, et al. A Series of Halogenated Heterodimeric Inhibitors of Prostate Specific Membrane Antigen (PSMA) as Radiolabeled Probes for Targeting Prostate Cancer. *Journal of Medicinal Chemistry.* 2009;52:347-357.

4. Horiuchi T, Chiba J, Uoto K, Soga T. Discovery of novel thieno[2,3-d]pyrimidin-4-yl hydrazone-based inhibitors of Cyclin D1-CDK4: Synthesis, biological evaluation, and structure-activity relationships. *Bioorganic & Medicinal Chemistry Letters.* 2009;19:305-308.

5. Zhou Z, Fahrni CJ. A fluorogenic probe for the copper(I)-catalyzed azide-alkyne ligation reaction: Modulation of the fluorescence emission via (3)(n,pi*)-(1)(pi,pi*) inversion. *Journal of the American Chemical Society.* 2004;126:8862-8863.

6. Bouvet V, Wuest M, Jans H-S, et al. Automated synthesis of [18F]DCFPyL via direct radiofluorination and validation in preclinical prostate cancer models. *EJNMMI Research.* 2016;6:40.

7. Mukherjee S, van der Donk WA. Mechanistic Studies on the Substrate-Tolerant Lanthipeptide Synthetase ProcM. *Journal of the American Chemical Society.* 2014;136:10450-10459.

8. Benešová M, Schäfer M, Bauder-Wüst U, et al. Preclinical Evaluation of a Tailor-Made DOTA-Conjugated PSMA Inhibitor with Optimized Linker Moiety for Imaging and Endoradiotherapy of Prostate Cancer. *Journal of Nuclear Medicine.* 2015;56:914-920.
